# Dynamics in Explicit Gradient Elasticity: Material Frame-Indifference, Boundary Conditions and Consistent Euler–Bernoulli Beam Theory

**DOI:** 10.3390/ma17081760

**Published:** 2024-04-11

**Authors:** Charalampos Tsakmakis, Carsten Broese, Stergios Alexandros Sideris

**Affiliations:** 1 Institute for Mechanics, Civil Engineering, Technical University of Darmstadt, Franziska-Braun-Str. 7, 64287 Darmstadt, Germany; 2 Ephorate of Antiquities of Lesvos, 81100 Mytilini, Greece; sterg_sid_87@hotmail.com

**Keywords:** Mindlin’s gradient elasticity, extensions of Hamilton’s principle, boundary conditions, material frame-indifference, acceleration terms, consistent Euler–Bernoulli beam theory

## Abstract

The paper is concerned with the boundary conditions of explicit gradient elasticity of Mindlin’s type in dynamics. It has been argued in an earlier paper that acceleration terms should not be present in the boundary tractions because of objectivity arguments. This is discussed in the present paper in more detail, and it is supplemented by assuming the validity of the principle of material frame indifference. Furthermore, new examples are discussed in order to illustrate that significant differences exist in the responses predicted by boundary tractions with and without acceleration terms.

## 1. Introduction

In classical elasticity, the stress tensor at a material point is a function of the strain tensor at that point. Such constitutive theories are called local. If the constitutive relations at a material point account, besides for the values of the state variables at the considered point, also for the values of the state variables in a neighborhood of that point, then the constitutive theory is called nonlocal. A simple possibility to capture nonlocality effects in the material response at a point is to incorporate in the theory the gradients of the state variables at that point. The arising theories are called gradient constitutive theories. Examples of gradient theories in solid mechanics are the gradient elasticity (see the references cited in [1]), the gradient plasticity (see the references cited in [2]) and phase field approaches to fracture (see the references cited in [3]), to name a few. It seems that systematic incorporation of gradient effects in elasticity, the present paper deals with, was initiated by the works of Korteweg in the year 1901 (see [4] and the references cited there) and Cosserat and Cosserat in the year 1909 (see [5] and the references cited there). The latter, accomplished with inertial terms, is nowadays known as micropolar elasticity [5]. The idea in this theory is to extend the notion of the classical continuum by attaching at any (macroscopic) material point a microcontinuum (microstructure), which is allowed to rotate like a rigid body. Whenever the microstructure is postulated to undergo homogenous deformations, the resulting continuum is denoted as micromorphic ([6], p. 5). The micromorphic elasticity introduced by Erringen (see [5], Section 7) and the microstructured elasticity theory introduced by Mindlin [7] are, in essence, the same and represent milestones in the development of nonclassical elasticity theories. Nonlocality effects in these theories are captured by the gradient of the deformation of the microcontinuum. The particular case where the microdeformation is set equal to the macrodeformation has been considered in [7] and is known as Mindlin’s gradient elasticity (the static version of this elasticity has been established by Toupin [8] without reference to a microstructure). Among others, Mindlin’s gradient elasticity can describe so-called length scale effects, which cannot be modeled by classical elasticity. Experimental evidence of such effects may be found in microbending tests of epoxy cantilever beams (see [9]), in vibration tests of nickel cantilever microbeams (see [10]), in pantographic structures (see [11]), in dispersion curves observed in metals, alloys and concrete (see [12]), in human calcaneus bones and in fluid saturated porous materials (cf. the references cited in [13,14]).

Mindlin’s gradient elasticity has been established by using a classical version of Hamilton’s variational principle. It obeys, for dynamic problems, traction boundary conditions, which include acceleration terms. This has been criticized in [15] by applying objectivity arguments. That means, as it has been stated in [15], that the boundary tractions are nonobjective and hence such boundary conditions are inacceptable physically. As a consequence, these authors proposed traction boundary conditions not including acceleration terms. Also, they calculated some uniaxial vibration examples in order to illustrate that the differences in the responses predicted by the theories with and without acceleration terms in the boundary tractions may be significant.

The present paper is concerned with two aspects of the traction boundary conditions in Mindlin’s gradient elasticity, which have not been addressed in [15]. The nonobjectivity of the boundary (contact) forces follows from the transformation rules for a change of observer. (The different types of forces used in classical mechanics and their objectivity properties are sketched in Section 2.2). However, as in the case of inertial forces (see Section 2.2), one could postulate the objectivity of contact forces, even if acceleration terms are present in these forces. This is the first aspect addressed in the paper. It is shown, as one might expect, that if the principle of material frame indifference is assumed for contact forces (and this is assumed in the present work), then to postulate objectivity of contact forces including acceleration terms is equivalent to the violation of this principle. These issues are discussed in Section 3.3. The second aspect addressed in the paper concerns the examples used to demonstrate the differences in predicted responses calculated with the two different types of boundary tractions. Nonvanishing nonclassical boundary tractions have been assumed to apply in the examples calculated in [15]. However, for the time being, it is not yet clear how to realize nonclassical boundary tractions. Therefore, homogenous nonclassical boundary tractions are assumed in the examples of the present paper, which seems to be more realistic. In addition, besides uniaxial loading of a bar, also vibrational loading of consistent Euler–Bernoulli beams, and besides traction-controlled also displacement-controlled loading histories are considered. When formulating boundary conditions, an appropriate way is to employ variational methods. For our purposes, especially, we find it convenient to employ extensions of Hamilton’s variational principle as explained in Section 2.1. (Note that bending of beams for Mindlin’s gradient elasticity in dynamics and within a consistent Euler–Bernoulli framework is studied for the first time in the present work). In summary, the present work completes the analysis provided in [15]. It does not investigate properties of a particular model, but rather provides an examination of the boundary tractions of the whole gradient elasticity of Mindlin’s type.

## 2. Basic Relations

### 2.1. Extensions of Hamilton’s Variational Principle

There are various extensions of Hamilton’s principle, which are in common use in solid mechanics. The extensions, which are of interest to our paper, can be discussed in a systematic way in the case of classical elasticity. Then,

(1) $$\delta \;\overset{t_{2}}{\underset{t_{1}}{\int }}\left[T-\Pi^{\;\left(i\right)}-\Pi^{\;\left(e\right)}\right]\mathrm{dt}\;=0\;,$$

is the standard form of the principle, where δ( ) is the variation of ( ) and t is the time. Further, T is the kinetic energy, which for classical elasticity reads

(2) $$T\equiv {Τ}^{\left(\mathrm{cl}\right)}=\overset{\;}{\underset{V}{\int }}\frac{1}{2}{\rho \dot{u}}_{i}{\dot{u}}_{i}\mathrm{dV}.$$

Here, ρ is the mass density and V is the range in three-dimensional Euclidean point space, occupied by the material body we consider. The boundary of V is ∂V  and has outward unit normal vector **n**. Unless explicitly stated otherwise, small deformations are assumed, all tensorial components are referred to a Cartesian coordinate system $\left\{x_{i}\right\},i=1,2,3$, and use is made of the summation convention. Material particles in V are identified with location vectors $x=x_{i}e_{i},$ where $\left\{e_{i}\right\}$ is the Cartesian coordinate basis. We write $\dot{\left(\;\right)}$ for the time derivative of ( ) and denote by $u_{i}$ the components of the displacement vector **u**. The variation $\delta u\left(x,t\right)$ is postulated to vanish everywhere at the initial and final times $t_{1},t_{2}$, while δu vanishes at all times in $\left[t_{1},t_{2}\right]$ on the boundary part $\partial V_{u}$, where displacement boundary conditions are prescribed. Conservative systems are addressed in Equation (1) and $\Pi^{\;\left(i\right)},\Pi^{\;\left(e\right)}$ are the potential energies of the internal and the external forces, respectively. Omitting volume forces and denoting by $\varphi \left(u,t\right)$ the potential per unit area for external forces, we have

(3) $$\Pi^{\;\left(e\right)}=\overset{\;}{\underset{\partial V}{\int }}\varphi \;\mathrm{dS}\;\Rightarrow \delta \;\overset{t_{2}}{\underset{t_{1}}{\int }}\Pi^{\;\left(e\right)}\;\mathrm{dt}\;=-\overset{t_{2}}{\underset{t_{1}}{\int }}\overset{\;}{\underset{\partial V_{p}}{\int }}P_{i}{\delta u}_{i}\;\mathrm{dS}\;\mathrm{dt}\;.$$

In the integral on the far-right side, $P_{i}:=-\frac{\partial \varphi }{\partial u_{i}}$, $\partial V_{P}$ denotes the part of ∂V where traction boundary conditions are given, and $\partial V_{u}\cup \partial V_{P}=\partial V,$ $\partial V_{u}\cap \partial V_{P}=\emptyset$. For classical elasticity, there exists a free energy pro unit volume ψ = ψ(**ε**), so that

(4) $$\Pi^{\;\left(i\right)}=\overset{\;}{\underset{V}{\int }}\psi \left(\varepsilon \right)\;\mathrm{dV}\;,$$

where **ε** is the strain tensor,

(5) $$\varepsilon_{\mathrm{ij}}:=\frac{1}{2}\left(\partial_{i}u_{j}+\partial_{j}u_{i}\right)\;.$$

Henceforward, we use the notation $\partial_{i}\left(\;\right):=\frac{\partial \left(\;\right)}{\partial x_{i}}\equiv \left(\;\right){,}_{x_{i}}$. Moreover, for classical elasticity, we define the Cauchy stress **Σ** through

(6) $$\Sigma_{\mathrm{ij}}:=\frac{\partial \psi }{\partial \varepsilon_{\mathrm{ij}}}\;.$$

Using these relations in Equation (1), after well-known manipulations, we arrive at

(7) $$\overset{t_{2}}{\underset{t_{1}}{\int }}\left[\;\overset{\;}{\underset{V}{\int }}\left(\rho {\ddot{u}}_{j}-\partial_{i}\Sigma_{\mathrm{ij}}\right){\delta u}_{j}\mathrm{dV}-\overset{\;}{\underset{\partial V_{p}}{\int }}\left(P_{j}-n_{i}\Sigma_{\mathrm{ij}}\right){\delta u}_{j}\mathrm{dS}\right]\;\mathrm{dt}\;=0\;.$$

With the aid of fundamental lemmas of calculus of variations (cf., e.g., [16], Section 2.4.), it can be proved that the balance of linear momentum

(8) $$\partial_{i}\Sigma_{\mathrm{ij}}=\rho {\ddot{u}}_{j},$$

together with the traction boundary conditions

(9) $$n_{i}\Sigma_{\mathrm{ij}}=P_{j}\;\mathrm{on}\;\partial V_{P}\times \left[t_{1},t_{2}\right]$$

are necessary and sufficient conditions for Equation (7).

The most frequent extension of the principle (1) is when a part of the external forces is conservative, with potential energy $\Pi^{\;\left(e\right)}$, and the remaining external forces are not conservative and expend virtual work ${\delta W}^{\;\left(e\right)}.$ Then the principle (1) is expressed in the form

(10) $$\delta \overset{t_{2}}{\underset{t_{1}}{\int }}\left[T-\Pi^{\;\left(i\right)}-\Pi^{\;\left(e\right)}\right]\mathrm{dt}+\overset{t_{2}}{\underset{t_{1}}{\int }}{\delta W}^{\;\left(e\right)}\mathrm{dt}=0\;.$$

Another extension of the principle (1) arises by introducing, as in d’Alembert’s principle, the inertial force $i:=-\;\rho \ddot{u}$ (cf. [17], Section 19), which expends virtual work ${\delta W}^{\;\left(\mathrm{inert}\right)}=\int_{V}^{\;}\;i_{j}{\delta u}_{j}\mathrm{dV}=-\int_{V}^{\;}\;\rho {\ddot{u}}_{j}{\delta u}_{j}\mathrm{dV}$. It can be seen that in this case $\delta \int_{t_{1}}^{t_{2}}T\;\mathrm{dt}=\int_{t_{1}}^{t_{2}}{\delta W}^{\;\left(\mathrm{inert}\right)}\mathrm{dt}$. Now, assume that a part of the inertial forces expends virtual work expressible as $\delta \int_{t_{1}}^{t_{2}}T\;\mathrm{dt}$, while the remainder of the inertial forces expend the work $\int_{t_{1}}^{t_{2}}{\delta W}^{\;\left(\mathrm{inert}\right)}\mathrm{dt}$. In this case, the principle (10) is extended as follows

(11) $$\delta \overset{t_{2}}{\underset{t_{1}}{\int }}\left[T-\Pi^{\;\left(i\right)}-\Pi^{\;\left(e\right)}\right]\mathrm{dt}+\overset{t_{2}}{\underset{t_{1}}{\int }}\left[{\delta W}^{\;\left(e\right)}+{\;\delta W}^{\;\left(\mathrm{inert}\right)}\right]\mathrm{dt}=0\;.$$

If only ${\delta W}^{\;\left(e\right)}$ and only ${\delta W}^{\;\left(\mathrm{inert}\right)}$ are considered, then the time integration in Equation (11) can be dropped out since

(12) $${\delta W}^{\;\left(\mathrm{inert}\right)}+{\delta W}^{\;\left(e\right)}-\delta \Pi^{\;\left(i\right)}=0$$

is the virtual work principle.

### 2.2. Material Frame-Indifference

Only in this section do the discussions refer to finite deformations. Following Liu [18] and Liu and Sampaio [19] (see also the references cited in these works), we define an observer or frame of reference to be a one-to-one map Φ assigning to a point (event) of space-time, a point on the product space of a three-dimensional Euclidean space and the set of real numbers (time axis). Let $x=x\left(X,t\right)$ be the motion of a material body relative to Φ, where X and x are the location vectors of a material particle in the reference and the actual configuration, respectively. The same motion with respect to another frame Φ* is denoted by $x^{*}=x^{*}\left(X^{*},t\right)$ and we have

(13) $$x^{*}=Q\left(t\right)x+c^{*}\left(t\right)\;,$$

where **Q** is an orthogonal transformation, **c** is a relative translation, and $t^{*}=t+a$, a = const.

Scalars s, vectors **v** and second-order tensors **T** are said to be Euclidean objective, or simply objective, if

(14) $$s^{*}=s,\;v^{*}=Qv,\;T^{*}=QTQ^{T}\;.$$

In these transformation rules, $A^{T}$ is the transpose of a second-order tensor **A** and $s^{*},v^{*},T^{*}$ are the quantities in the frame Φ* corresponding to s, **v**, **T** in the frame Φ, respectively.

In classical mechanics, there are different classifications of forces. On the one hand, forces are divided into those that obey a response law and those that cannot be determined by response laws but are calculated from balance laws and boundary conditions. The existence of the second type of forces arises from imposed geometrical conditions. Examples of the first type of forces are spring forces, obeying an elasticity law, and inertial forces, obeying, e.g., the response law $-\rho \ddot{u}$ with respect to an inertial reference frame. Examples of the second type of forces are reaction forces due to supports, the geometrical constraint being, e.g., no displacement, or the pressure in incompressible continua, the geometrical constraint being isochoric deformations. On the other hand, forces in continuum mechanics are divided into contact, body and inertial forces. A fundamental postulate in classical mechanics is that all forces mentioned above are assumed to be objective.

Now let U be a set of state and kinematical variables and suppose that an objective quantity J obeys a response law $J=f_{\Phi }\left(U\right)\;$ in the frame Φ. For simplicity, $f_{\Phi }$ is supposed here to be a function, but in the general case it may be a functional. Generally, response functions may be observer-dependent, so that $J^{*}=f_{\Phi *}\;\left(U^{*}\right)$. The response function is said to be observer-independent, or material frame-indifferent (see [6,7,8], Section 19), or simply frame-indifferent, if it has the same form in all frames, i.e., $f_{\Phi }\left(\;\right)=f_{\Phi *}\left(\;\right)$. When formulating constitutive laws for ordinary stresses, the response functions are required to be frame-indifferent, which imposes restrictions on the form of the functions that are to be designed. On the contrary, the form of the response functions of inertial forces is supposed to be known with respect to an inertial frame. The forms of the response functions of the inertial forces in other, noninertial frames are derived on the basis of the transformation rules (13) and (14), imposed by the postulated objectivity of inertial forces, and are generally dependent on the observer (cf. [20]). This is a consequence of the unique feature characteristics of inertial forces.

### 2.3. A Simple Model of Explicit Gradient Elasticity

Consider material bodies which, for omitting body forces, are described by balance laws of linear and angular momentum, so that at any point in V the field equations

(15) $$\partial_{j}\Sigma_{\mathrm{jk}}+i_{k}=0\;,$$

(16) $$\Sigma_{\mathrm{jk}}=\Sigma_{\mathrm{kj}}\;$$

apply. As in Equation (8), **Σ** is the Cauchy stress tensor, while **i** is the inertial force. In Mindlin’s gradient elasticity (see [7]), **i** is decomposed into classical and nonclassical parts,

(17) $$i=i^{\left(\mathrm{cl}\right)}+i^{\left(\mathrm{noncl}\right)}\;,\;$$

with

(18) $$i_{k}^{\left(\mathrm{cl}\right)}:=-\;\rho \;{\ddot{u}}_{k}\;.$$

For simplicity,

(19) $$i_{k}^{\left(\mathrm{noncl}\right)}:=\gamma \partial_{p}\partial_{p}\;{\ddot{u}}_{k}=\;\gamma \;\Delta \;{\ddot{u}}_{k}$$

is chosen in the present paper, where Δ is the Laplacian operator and γ = const. is a material parameter. The inertial law (19) corresponds to the “isotropic case” in Mindlin’s work (see p. 70 in [7]).

A simple gradient elasticity model arises by assuming the free energy per unit volume ψ to be given by

(20) $$\psi =\psi \left(\varepsilon ,k\right)=\frac{1}{2}\varepsilon_{\mathrm{jk}}K_{\mathrm{jkmn}}\varepsilon_{\mathrm{mn}}+\frac{1}{2}l^{2}k_{\mathrm{ijk}}K_{\mathrm{jkmn}}{\;k}_{\mathrm{imn}}\;,$$

where

(21) $$k_{\mathrm{ijk}}:=\partial_{i}\varepsilon_{\mathrm{jk}}$$

is the gradient of **ε**. Further, K is an anisotropic fourth-order elasticity tensor exhibiting the symmetries $K_{\mathrm{jkmn}}=K_{\mathrm{kjmn}}=K_{\mathrm{mnjk}}$ and l = const. is an internal material length. According to the gradient elasticity Form II of Mindlin’s theory (see [7]), **Σ** obeys the constitutive law

(22) $$\Sigma_{\mathrm{jk}}:=\tau_{\mathrm{jk}}-\partial_{i}\mu_{\mathrm{ijk}}\;,$$

(23) $$\tau_{\mathrm{jk}}=\tau_{\mathrm{kj}}:=\frac{\partial \psi \left(\varepsilon ,k\right)}{\partial \varepsilon_{\mathrm{jk}}}=K_{\mathrm{jkmn}}{\;\varepsilon }_{\mathrm{mn}}\;,\;$$

(24) $$\mu_{\mathrm{ijk}}=\mu_{\mathrm{ikj}}:=\frac{\partial \psi \left(\varepsilon ,k\right)}{\partial k_{\mathrm{ijk}}}=l^{2}K_{\mathrm{jkmn}}\left(\partial_{i}\varepsilon_{\mathrm{mn}}\right)\;,$$

where **τ** is a classical, second-order stress tensor of Cauchy type and **μ** is a nonclassical, third-order stress tensor. From Equations (22)–(24),

(25) $$\Sigma_{\mathrm{jk}}=K_{\mathrm{jkmn}}\varepsilon_{\mathrm{mn}}-l^{2}K_{\mathrm{jkmn}}{\Delta \varepsilon }_{\mathrm{mn}}\;.$$

To our knowledge, this constitutive law, with K being an isotropic elasticity tensor, has been proposed for the first time in Altan and Aifantis [21]. In Broese et al. [22], the gradient elasticity law (25) has been interpreted to represent the gradient elasticity analog of the Kelvin viscoelastic solid. In the following, we shall denote it as Kelvin-Gradient elasticity Model (KG-Model).

To accomplish the theory, concomitant boundary conditions for the field equations remain to be formulated. As mentioned in the introduction, an appropriate way to establish these is to invoke variational principles.

## 3. Gradient Elasticity in the Setting of Hamilton’s Principle

### 3.1. Variational Formulation of the Field Equations

After multiplying Equation (15) by the virtual displacement δ$u_{k}$, integrating over V and using partial integration, we obtain

(26) $$\overset{\;}{\underset{V}{\int }}\partial_{j}\left(\Sigma_{\mathrm{jk}}{\delta u}_{k}\right)\mathrm{dV}-\overset{\;}{\underset{V}{\int }}\Sigma_{\mathrm{jk}}\delta \varepsilon_{\mathrm{jk}}\;\mathrm{dV}+\overset{\;}{\underset{V}{\int }}i_{k}{\delta u}_{k}\mathrm{dV}=0\;.$$

Next, we replace $\Sigma_{\mathrm{jk}}$ in the second integral with the aid of the constitutive law (22),

(27) $$\overset{\;}{\underset{V}{\int }}\partial_{j}\left(\Sigma_{\mathrm{jk}}{\delta u}_{k}\right)\mathrm{dV}-\overset{\;}{\underset{V}{\int }}\tau_{\mathrm{jk}}\delta \varepsilon_{\mathrm{jk}}\;\mathrm{dV}+\overset{\;}{\underset{V}{\int }}(\partial_{m}{\;\mu }_{\mathrm{mjk}})\;\delta \varepsilon_{\mathrm{jk}}\;\mathrm{dV}+\overset{\;}{\underset{V}{\int }}i_{k}{\delta u}_{k}\mathrm{dV}=0\;,$$

or equivalently

(28) $$\overset{\;}{\underset{V}{\int }}\partial_{j}\left[\Sigma_{\mathrm{jk}}{\delta u}_{k}+\mu_{\mathrm{jik}}\left(\partial_{i}{\delta u}_{k}\right)\right]\mathrm{dV}-\overset{\;}{\underset{V}{\int }}[\tau_{\mathrm{jk}}\delta \varepsilon_{\mathrm{jk}}+\mu_{\mathrm{mjk}}\;\delta \left(\partial_{m}\varepsilon_{\mathrm{jk}}\right)]\;\mathrm{dV}\;+\overset{\;}{\underset{V}{\int }}i_{k}{\delta u}_{k}\mathrm{dV}=0\;.$$

We recall from Equations (20), (23) and (24), that the second integral is the virtual work of internal forces, i.e.,

(29) $$\overset{\;}{\underset{V}{\int }}\partial_{j}\left[\Sigma_{\mathrm{jk}}{\delta u}_{k}+\mu_{\mathrm{jik}}\left(\partial_{i}{\delta u}_{k}\right)\right]\mathrm{dV}-{\delta \Pi }^{\;\left(i\right)}+\overset{\;}{\underset{V}{\int }}i_{k}{\delta u}_{k}\mathrm{dV}=0\;,$$

with (cf. Equation (4))

(30) $$\Pi^{\;\left(i\right)}:=\overset{\;}{\underset{V}{\int }}\psi \left(\varepsilon ,\;k\right)\;\mathrm{dV}\;.$$

In order to recast the virtual work expended by the inertial force **i**, we recall from Equations (17)–(19) that

(31) $$\overset{\;}{\underset{V}{\int }}i_{k}{\delta u}_{k}\mathrm{dV}\;=-\overset{\;}{\underset{V}{\int }}\;\rho {\ddot{u}}_{k}{\;\delta u}_{k}\;\mathrm{dV}\;+\overset{\;}{\underset{V}{\int }}i_{k}^{\left(\mathrm{noncl}\right)}{\;\delta u}_{k}\;\mathrm{dV}\;\;=-\frac{d}{\mathrm{dt}}\;\overset{\;}{\underset{V}{\int }}{\rho \dot{u}}_{k}{\;\delta u}_{k}\;\mathrm{dV}\;+\delta \overset{\;}{\underset{V}{\int }}\frac{1}{2}{\rho \dot{u}}_{k}{\dot{u}}_{k}\;\mathrm{dV}\;+\overset{\;}{\underset{V}{\int }}i_{k}^{\left(\mathrm{noncl}\right)}{\;\delta u}_{k}\;\mathrm{dV}\;=-\frac{d}{\mathrm{dt}}\overset{\;}{\underset{V}{\int }}{\rho \dot{u}}_{k}{\;\delta u}_{k}\;\mathrm{dV}+{\delta T}^{\left(\mathrm{cl}\right)}+\overset{\;}{\underset{V}{\int }}i_{k}^{\left(\mathrm{noncl}\right)}{\;\delta u}_{k}\;\mathrm{dV},$$

where $T^{\left(\mathrm{cl}\right)}$ is the classical kinetic energy defined in Equation (2). Thus, by taking the time integral between $t_{1}$ and $t_{2}$ of Equation (29), and using the result (31), we find that

(32) $$\overset{t_{2}}{\underset{t_{1}}{\int }}\overset{\;}{\underset{V}{\int }}\;\partial_{j}\left[\Sigma_{\mathrm{jk}}{\delta u}_{k}+\mu_{\mathrm{jik}}\left(\partial_{i}{\delta u}_{k}\right)\right]\mathrm{dVdt}-\delta \overset{t_{2}}{\underset{t_{1}}{\int }}\Pi^{\;\left(i\right)}\;\mathrm{dt}\;+\delta \overset{t_{2}}{\underset{t_{1}}{\int }}T^{\left(\mathrm{cl}\right)}\;\mathrm{dt}\;\;+\overset{t_{2}}{\underset{t_{1}}{\int }}\overset{\;}{\underset{V}{\int }}i_{k}^{\left(\mathrm{noncl}\right)}{\;\delta u}_{k}\;\mathrm{dVdt}=0\;.$$

Now, there are two ways to recast the volume integral of the last term. One is according to Mindlin [7] and leads to boundary conditions involving acceleration terms, whereas the second way, according to Broese et al. [15], leads to boundary conditions in which acceleration terms are not present.

### 3.2. Gradient Elasticity with Acceleration Terms Present in the Boundary Conditions

The aim of Mindlin [7] was to bring Equation (32) to a form corresponding to Equation (1), or to the more general Equation (10). To achieve this, the last term in Equation (32) is rewritten using the definition (19):

(33) $$\overset{t_{2}}{\underset{t_{1}}{\int }}\overset{\;}{\underset{V}{\int }}i_{k}^{\left(\mathrm{noncl}\right)}{\;\delta u}_{k}\;\mathrm{dVdt}\;=\overset{t_{2}}{\underset{t_{1}}{\int }}\overset{\;}{\underset{V}{\int }}\gamma \left(\partial_{j}\partial_{j}{\ddot{u}}_{k}\right){\delta u}_{k}\;\mathrm{dVdt}\;\;=\overset{t_{2}}{\underset{t_{1}}{\int }}\overset{\;}{\underset{V}{\int }}\partial_{j}\left[\gamma \left(\partial_{j}{\ddot{u}}_{k}\right){\delta u}_{k}\right]\mathrm{dVdt}\;-\overset{{\;t}_{2}\;}{\underset{t_{1}}{\int }}\overset{\;}{\underset{V}{\int }}\gamma \left(\partial_{j}{\ddot{u}}_{k}\right)\;\delta (\partial_{j}u_{k})\;\mathrm{dVdt}\;\;=\overset{t_{2}}{\underset{t_{1}}{\int }}\overset{\;}{\underset{V}{\int }}\partial_{j}\left[\gamma \left(\partial_{j}{\ddot{u}}_{k}\right){\delta u}_{k}\right]\mathrm{dVdt}\;-\overset{t_{2}}{\underset{t_{1}}{\int }}\frac{d}{\mathrm{dt}}\left[\;\overset{\;}{\underset{V}{\int }}\gamma \left(\partial_{j}{\overset{\cdot }{u}}_{k}\right)\;\delta (\partial_{j}u_{k})\;\mathrm{dV}\right]\;\mathrm{dt}\;\;+\overset{t_{2}}{\underset{t_{1}}{\int }}\overset{\;}{\underset{V}{\int }}\gamma \;\left(\partial_{j}{\overset{\cdot }{u}}_{k}\right)\;\delta \left(\partial_{j}{\overset{\cdot }{u}}_{k}\right)\;\mathrm{dV}\;\mathrm{dt}\;.$$

On the far-right side, the second integral vanishes since $\partial_{j}{\delta u}_{k}\left(t_{1}\right)=\partial_{j}{\delta u}_{k}\left(t_{2}\right)\equiv 0$ in V, while the last integral can be represented by the variation of nonclassical kinetic energy

(34) $${\bar{T}}^{\left(\mathrm{noncl}\right)}:=\overset{\;}{\underset{V}{\int }}\frac{1}{2}\gamma \;\left(\partial_{j}{\overset{\cdot }{u}}_{k}\right)\;\left(\partial_{j}{\overset{\cdot }{u}}_{k}\right)\mathrm{dV}\;.$$

Hence, Equation (33) implies

(35) $$\overset{t_{2}}{\underset{t_{1}}{\int }}\overset{\;}{\underset{V}{\int }}i_{k}^{\left(\mathrm{noncl}\right)}{\;\delta u}_{k}\;\mathrm{dVdt}=\overset{t_{2}}{\underset{t_{1}}{\int }}\overset{\;}{\underset{V}{\int }}\partial_{j}\left[\gamma \left(\partial_{j}\;{\ddot{u}}_{k}\right){\delta u}_{k}\right]\mathrm{dVdt}+\delta \overset{t_{2}}{\underset{t_{1}}{\int }}{\bar{T}}^{\left(\mathrm{noncl}\right)}\;\mathrm{dt}\;,$$

and by inserting into Equation (32), we obtain

(36) $$\delta \overset{t_{2}}{\underset{t_{1}}{\int }}\left[T^{\left(\mathrm{cl}\right)}+{\bar{T}}^{\left(\mathrm{noncl}\right)}-\Pi^{\;\left(i\right)}\right]\mathrm{dt}\;+\overset{t_{2}}{\underset{t_{1}}{\int }}\overset{\;}{\underset{V}{\int }}\;\partial_{j}\left[\left(\Sigma_{\mathrm{jk}}+\gamma \left(\partial_{j}\;{\ddot{u}}_{k}\right)\right){\delta u}_{k}+\mu_{\mathrm{jik}}\left(\partial_{i}{\delta u}_{k}\right)\right]\mathrm{dVdt}\;=0\;.\;$$

This has been interpreted by Mindlin to suggest defining a total Cauchy stress tensor $\Sigma^{\left(t\right)}$ involving acceleration terms,

(37) $$\Sigma_{\mathrm{jk}}^{\left(t\right)}:=\Sigma_{\mathrm{jk}}+\gamma \left(\partial_{j}{\ddot{u}}_{k}\right)\;,$$

a virtual work of external forces

(38) $${\delta \bar{W}}^{\left(e\right)}:=\overset{\;}{\underset{\partial V}{\int }}n_{j}\left(\Sigma_{\mathrm{jk}}^{\left(t\right)}{\delta u}_{k}+\mu_{\mathrm{jik}}\left(\partial_{i}{\delta u}_{k}\right)\right)\mathrm{dS}\;,\;$$

and the total kinetic energy

(39) $$\bar{T}:={\;T}^{\left(\mathrm{cl}\right)}+{\bar{T}}^{\left(\mathrm{noncl}\right)}\;.$$

Then, Equation (36) becomes

(40) $$\delta \overset{t_{2}}{\underset{t_{1}}{\int }}\left[\bar{T}-\Pi^{\;\left(i\right)}\right]\mathrm{dt}\;+\overset{t_{2}}{\underset{t_{1}}{\int }}{\delta \bar{W}}^{\left(e\right)}\mathrm{dt}\;=0\;,\;$$

which is of the form (10).

To accomplish Mindlin’s approach, the surface integral ${\delta \bar{W}}^{\left(e\right)}$ must be resolved further, since the gradient $\partial_{j}{\delta u}_{k}$ is not independent of ${\delta u}_{k}$ on ∂V. After lengthy and elaborate algebraic manipulations, it can be proved (see [7,15]) that

(41) $${\delta \bar{W}}^{\left(e\right)}=\overset{\;}{\underset{\partial V}{\int }}\left[{\bar{P}}_{k}{\;\delta u}_{k}+{\bar{R}}_{k}\;\left({D\delta u}_{k}\right)\right]\mathrm{dS}\;,$$

where $\bar{P}$ and $\bar{R}$ are classical and nonclassical traction vectors, respectively, defined by

(42) $${\bar{P}}_{k}:=n_{j}\Sigma_{\mathrm{jk}}^{\left(t\right)}-D_{j}\left(n_{i}\mu_{\mathrm{ijk}}\right)+\left(D_{l}n_{l}\right)\left(n_{i}n_{j}\mu_{\mathrm{ijk}}\right)\;=n_{j}\Sigma_{\mathrm{jk}}+\gamma \left(\partial_{j}{\ddot{u}}_{k}\right)n_{j}-D_{j}\left(n_{i}\mu_{\mathrm{ijk}}\right)+\left(D_{l}n_{l}\right)\left(n_{i}n_{j}\mu_{\mathrm{ijk}}\right)\;,$$

(43) $${\bar{R}}_{k}:=n_{i}n_{j}\mu_{\mathrm{ijk}}\;.\;$$

For a function f(**x**,t), the normal derivative Df(**x**,t) and the surface derivative $D_{i}f\left(x,t\right)$ are defined through

(44) $$\mathrm{Df}:=n_{l}\partial_{l}f\;,{\;D}_{i}f:=\partial_{i}f\;-n_{i}\mathrm{Df}\;.$$

This way, ${\delta \bar{W}}^{\left(e\right)}$ is interpreted as the virtual work expended by the tractions $\bar{P}$ and $\bar{R}$, and Equation (40) is assumed to be the appropriate form of Hamilton’s principle for the considered material. Since now δ**u** and Dδ**u** are independent of each other on ∂V, the adjoint boundary conditions suggested by Equation (41) are

(45) $${\mathrm{either}\;\bar{P}}_{k}{\;\mathrm{or}\;u}_{k}\;\mathrm{and}\;$$

(46) $${\mathrm{either}\;\bar{R}}_{k}{\;\mathrm{or}\;\mathrm{Du}}_{k}\;$$

have to be prescribed on ∂V.

Altogether, for the gradient elasticity based on the KG-Model, Equations (15)–(19) are the governing equations of motion and Equations (45) and (46) are the adjoint boundary conditions proposed by Mindlin. The remarkable feature in the traction boundary condition (45) is the presence of acceleration terms in $\bar{P}$ (see Equation (42). Moreover, $\Sigma^{\left(t\right)}$ cannot be a proper classical Cauchy stress tensor, for it is generally not symmetric (because of the presence of the acceleration term in Equation (37)).

### 3.3. Gradient Elasticity without Acceleration Terms Present in the Boundary Conditions

As mentioned in Section 2.2, contact, body and inertial forces are postulated to be objective and, in addition, constitutive functions of ordinary Cauchy stresses are required to be frame-indifferent. It can be proved, that the presence of acceleration terms in Equation (42) renders the traction vector **P** to be non objective. If someone would get the idea to postulate **P** as objective, as in the case of inertial forces, then one would have to conclude that the constitutive function of stress $\Sigma^{\left(t\right)}$ in Equation (37) will not be frame-indifferent. Either way, we believe that the presence of acceleration terms in boundary conditions for contact forces is physically not acceptable and this is, in principle, the criticism made by Broese et al. [15]. These authors proposed an alternative but not equivalent definition for the virtual work expended by external forces. Considering inertial forces to have the nature of conventional body forces (see [17], Section 21), they proposed to interpret/rewrite Equation (32) as follows.

Let ${\delta \hat{W}}^{\left(\mathrm{inert},\mathrm{noncl}\right)}$ denote the virtual work of the inertial force $i^{\left(\mathrm{noncl}\right)}$,

(47) $${\delta \hat{W}}^{\left(\mathrm{inert},\mathrm{noncl}\right)}:=\overset{\;}{\underset{V}{\int }}i_{k}^{\left(\mathrm{noncl}\right)}{\delta u}_{k}\mathrm{dV}=\overset{\;}{\underset{V}{\int }}\gamma \left(\Delta {\ddot{u}}_{k}\right){\;\delta u}_{k}\mathrm{dV},$$

and reformulate Equation (32) in the form

(48) $$\;\delta \overset{t_{2}}{\underset{t_{1}}{\int }}\left(T^{\left(\mathrm{cl}\right)}-{\;\Pi }^{\;\left(i\right)}\right)\;\mathrm{dt}\;+\overset{t_{2}}{\underset{t_{1}}{\int }}\left({\delta \hat{W}}^{\left(e\right)}+{\delta \hat{W}}^{\left(\mathrm{inert},\mathrm{noncl}\right)}\right)\;\mathrm{dt}\;=0,$$

where

(49) $${\delta \hat{W}}^{\left(e\right)}:=\overset{\;}{\underset{V}{\int }}\partial_{j}\left(\Sigma_{\mathrm{jk}}{\delta u}_{k}+\mu_{\mathrm{jik}}\partial_{i}\left({\delta u}_{k}\right)\right)\;\mathrm{dV}.$$

We view Equation (48) to be of the form (11), and recognize ${\delta \hat{W}}^{\left(e\right)}$ as the proper virtual work of the external forces and $\Sigma_{\mathrm{jk}}=\Sigma_{\mathrm{kj}}$ as the proper Cauchy stress tensor, which should enter into the boundary tractions. By performing similar calculations as for the transition from Equation (38) to Equations (41)–(43), we can introduce proper boundary tractions $\hat{P}$ and $\hat{R}$, so that

(50) $${\delta \hat{W}}^{\left(e\right)}=\overset{\;}{\underset{\partial V}{\int }}\left[{\hat{P}}_{k}{\delta u}_{k}+{\hat{R}}_{k}\delta \left({\mathrm{Du}}_{k}\right)\right]\mathrm{dS},$$

where (cf. [15,16])

(51) $${\hat{P}}_{k}:=n_{j}\Sigma_{\mathrm{jk}}-D_{j}\left(n_{i}\mu_{\mathrm{ijk}}\right)+\left(D_{l}n_{l}\right)\left(n_{i}n_{j}\mu_{\mathrm{ijk}}\right),$$

(52) $${\hat{R}}_{k}:=n_{i}n_{j}\mu_{\mathrm{ijk}}.$$

Accordingly, the proper boundary conditions read

(53) $${\mathrm{either}\;\hat{P}}_{k}{\;\mathrm{or}\;u}_{k}\;\mathrm{and}\;$$

(54) $$\mathrm{either}\;{\hat{R}}_{k}{\;\mathrm{or}\;\mathrm{Du}}_{k}\;$$

have to be prescribed on ∂V. No acceleration terms are now involved in the boundary condition for classical traction, but otherwise, the governing equations of motion are the same as in Equations (15)–(19).

## 4. Consistent Euler–Bernoulli Beam Theory in Dynamics

### 4.1. Main Assumptions

The traditional Euler–Bernoulli beam theory relies upon two fundamental assumptions (see [23], Sections 5.1 and 5.4.2 and [13], p. 90): (1) The material response is isotropic elastic. (2) Plane cross sections of the beam remain plane and perpendicular to the deformed beam axis and the shape of the cross sections does not change (no deformation of cross sections). It is obvious, that no deformation of cross sections is not consistent with isotropic elasticity. This inconsistency is reflected by the fact that the elasticity law is satisfied in local form, but, e.g., the equilibrium equations in statics are not satisfied in local form (see [24] and the references cited there).

To overcome this problem, Sideris and Tsakmakis [24] (see also [25]) proposed to drop the assumption of isotropy and instead suppose transversal isotropic material behavior subject to geometrical constraints. This way, they obtained consistent Euler–Bernoulli beam theories. The main kinematical and constitutive equations of this approach for the KG-Model can be summarized as follows (see [24]).

**Figure 1:**
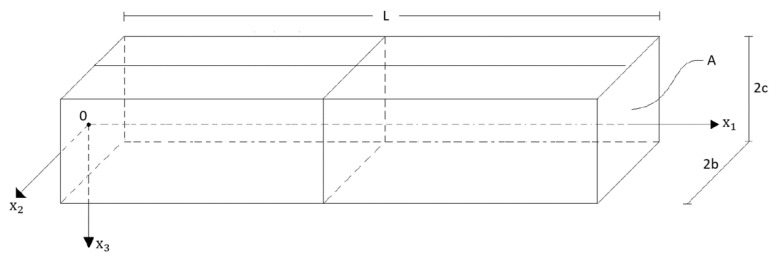
Rectangular beam of length L, width 2b and height 2c.

Consider the beam in Figure 1, which is of length L, and constant cross section A, with width 2b and height 2c. The origin of the Cartesian coordinate system {$x_{i}\}$ is located on the left boundary plane, the $x_{1}-x_{3}$ plane is a symmetry plane, and the $x_{1}-$ axis is the centroidal axis of the beam. The beam might be subject to a transverse load, which acts in the $x_{1}-x_{3}$ plane at $x_{3}=\pm \;c$, and to axial loads, which act on the boundary planes $x_{1}=0$ and $x_{1}=L$. In addition, problem-specific boundary/reaction forces will apply. The material response is assumed to be transverse isotropic with vanishing in-plane Poisson ratios and subject to internal constraints. All assumptions together cause conditions of plane strain and plane stress with **u**, **ε**, **Σ** being functions only of $x_{1}$, $x_{3}$ and

(55) $$u_{2}=\varepsilon_{2i}=\Sigma_{2i}=0.$$

The remaining components of the displacement vector and the strain tensor are given by

(56) $$u_{1}=U\left(x_{1},t\right)-w^{\prime }\left(x_{1},t\right)x_{3},{\;u}_{2}\equiv 0,{\;u}_{3}=w\left(x_{1},t\right),$$

(57) $$\varepsilon_{11}=U^{\prime }-{\;wx^{\prime \prime }}_{3},{\;\varepsilon }_{33}=\varepsilon_{13}=0,$$

where U is axial displacement, w$\left(x_{1},t\right)$ is the deflection curve and $f^{\prime }\left(x_{1},t\right)$ is the derivative of the function $f\left(x_{1},t\right)$ with respect to $x_{1}$. The assumed geometrical constraints do not allow to determine every stress component from the elasticity laws (22)–(24). In fact, we have (see [24,25])

(58) $$\Sigma_{11}={E\varepsilon }_{11}-\mu_{111}^{\prime }=\;E\;\left(U^{\prime }-w^{\prime }x_{3}\right)-l^{2}\;E\;\left(U^{\prime \prime \prime }-w^{⁗}x_{3}\;\right),$$

(59) $$\tau_{11}={E\varepsilon }_{11}=E\left(U^{\prime }-{w^{\prime \prime }\;x}_{3}\right),$$

(60) $$\mu_{111}=l^{2}{E\varepsilon }_{11}^{\prime }=l^{2}E\left(U^{\prime \prime }-{w^{\prime \prime \prime }\;x}_{3}\right),$$

(61) $$\mu_{311}=l^{2}E\;\left(\;\partial_{3}{\;\varepsilon }_{11}\right)=-l^{2}E\;w^{\prime \prime },$$

$$\tau_{33},\tau_{13},\mu_{i33},\Sigma_{33},\Sigma_{13}:\mathrm{not}\;\mathrm{determinable}\;\mathrm{by}\;\mathrm{constitutive}\;\mathrm{law},$$

while ψ in Equation (20) becomes

(62) $$\psi =\frac{1}{2}{E\varepsilon }_{11}^{2}+\frac{l^{2}}{2}E\;{\left(\partial_{1}\varepsilon_{11}\right)}^{2}+\frac{l^{2}}{2}E{\left(\partial_{3}\varepsilon_{11}\right)}^{2}.$$

Every other stress component is vanishing, while E is Young’s modulus in $x_{1}$-direction. Only statics has been addressed in Sideris and Tsakmakis [24] and Broese et al. [25], and therefore only equilibrium equations have come into play in these works. However, the present paper is concerned with dynamics and hence the equations of motion (15)–(19) must be used. For the plane stress and plane strain conditions set up above, these reduce to the two equations

(63) $$\partial_{1}\Sigma_{11}+\partial_{3}\Sigma_{13}=\rho {\ddot{u}}_{1}-\;\gamma {\ddot{u}}_{1}^{\prime \prime }\;,$$

(64) $$\partial_{1}\Sigma_{13}+\partial_{3}\Sigma_{33}=\rho {\ddot{u}}_{3}-\;\gamma {\ddot{u}}_{3}^{\prime \prime }\;.$$

In the remainder of the paper, we shall use frequently the definitions

(65) $$f_{1}\left(x_{1}\right):=\mathrm{EA}\left(U^{\prime }-l^{2}U^{\prime \prime \prime }\right),{\;f}_{2}\left(x_{1}\right):=l^{2}\mathrm{EA}U^{\prime \prime },$$

(66) $$f_{3}\left(x_{1}\right):=-\left(\mathrm{EI}+l^{2}\mathrm{EA}\right)w^{\prime \prime }+l^{2}\mathrm{EI}w^{⁗}\;,{\;f}_{4}\left(x_{1}\right):=l^{2}\mathrm{EI}w^{\prime \prime \prime }.$$

Furthermore, we denote by I the moment of inertia and we notice that, with respect to the chosen coordinate system, we have

(67) $$I=\overset{\;}{\underset{A}{\int }}x_{3}^{2}\mathrm{dS}\;,\;\overset{\;}{\underset{A}{\int }}x_{3}\mathrm{dS}=0.$$

When solving equations of the form (63) and (64), the effort can be reduced considerably by simplifying the problem using section and resultant forces. The resulting equations correspond to a one-dimensional continuum, which is bounded by the points $x_{1}=0$, $x_{1}=L$. Points $x_{1}\in \left(0,L\right)$ are interior points and traction boundary conditions for the three-dimensional body on the planes $x_{3}=\pm c$ are accounted for at any $x_{1}$ of the one-dimensional beam continuum as resultants of these tractions. These resultants may be viewed as body forces for the one-dimensional continuum acting on interior points $x_{1}.$ Thus, the forces which act on the one-dimensional beam, or any sub-body of it, are section forces on the boundaries and resultant (body) forces distributed along $x_{1}$. A convenient way to introduce these forces is to invoke variational methods.

### 4.2. Variational Methods of Gradient Elastic Euler-Bernoulli Beams

At the beginning of Section 3.3, it has been argued that the presence of acceleration terms in the traction boundary conditions is physically unacceptable. Nevertheless, for comparison, we shall elaborate variational formulations of gradient elastic Euler–Bernoulli beams for both cases of the KG-Model with and without acceleration terms in the boundary tractions.

#### 4.2.1. Hamilton’s Principle for the Case Where Acceleration Terms Are Present in the Traction Boundary Conditions

##### **Approach Based on Hamilton’s Principle Equation (40)** 

It is instructive first to evaluate Hamilton’s principle (40) by specializing ${\delta \bar{W}}^{\left(e\right)}$, $\Pi^{\;\left(i\right)},\bar{T}$ to the assumed Euler–Bernoulli kinematics. Several algebraic manipulations are similar to those elaborated in Sideris and Tsakmakis [24] and Broese et al. [25], and therefore they will be only sketched briefly here.

Using steps quite similar to those in Sideris and Tsakmakis [24] (see also [25]), it can be seen that ${\delta \bar{W}}^{\left(e\right)}$ in Equations (41)–(43) can be expressed in terms of section forces $\bar{N},$ $\bar{H}$, $\bar{V}$, $\bar{M}$, $\bar{m}$ and resultant forces $\bar{p}$, $\bar{q}$ as follows:

(68) $${\delta \bar{W}}^{\left(e\right)}=\overset{\;}{\underset{\partial V}{\int }}\left[{\bar{P}}_{k}{\delta u}_{k}+{\bar{R}}_{k}\left({D\delta u}_{k}\right)\right]\mathrm{dS}\;\;={\left[\bar{N}\delta U\right]}_{x_{1}=0}^{x_{1}=L}+{\left[\bar{H}\delta U^{\prime }\right]}_{x_{1}=0}^{x_{1}=L}+{\left[\bar{V}\delta w\right]}_{x_{1}=0}^{x_{1}=L}+{\left[\bar{M}\delta \left(-w^{\prime }\right)\right]}_{x_{1}=0}^{x_{1}=L}\;+{\left[\bar{m}\delta \left(-w^{\prime \prime }\right)\right]}_{x_{1}=0}^{x_{1}=L}+\overset{L}{\underset{0}{\int }}{\bar{p}\delta U\;\mathrm{dx}}_{1}+\overset{L}{\underset{0}{\int }}{\bar{q}\delta w\;\mathrm{dx}}_{1}.$$

The potential of the internal forces can be determined from the free energy in Equation (62),

(69) $$\Pi^{\;\left(i\right)}=\overset{\;}{\underset{V}{\int }}\frac{1}{2}E\left(\varepsilon_{11}^{2}+l^{2}{\left(\partial_{1}\varepsilon_{11}\right)}^{2}+l^{2}{\left(\partial_{3}\varepsilon_{11}\right)}^{2}\right)\mathrm{dV},$$

or equivalently, by virtue of Equation (57),

(70) $$\Pi^{\;\left(i\right)}=\overset{\;}{\underset{V}{\int }}\frac{1}{2}E\left[{\left(U^{\prime }-{w^{\prime \prime }x}_{3}\right)}^{2}+l^{2}{\left(U^{\prime \prime }-{w^{\prime \prime \prime }x}_{3}\right)}^{2}+l^{2}{\left(w^{\prime \prime }\right)}^{2}\right]\mathrm{dV}.$$

If U ≡0, then the potential (70) reduces to the one, which was essentially the starting point in the work of Lazopoulos and Lazopoulos [26]. After lengthy manipulations and repeatedly applying partial integration, we find that

(71) $${\delta \Pi }^{\;\left(i\right)}={\left[f_{1}\delta U\right]}_{x_{1}=0}^{x_{1}=L}+{\left[f_{2}\delta \;U^{\prime }\right]}_{x_{1}=0}^{x_{1}=L}+{\left[f_{3}^{\prime }\delta w\right]}_{x_{1}=0}^{x_{1}=L}+{\left[f_{3}\;\delta \left(-w^{\prime }\right)\right]}_{x_{1}=0}^{x_{1}=L}\;\;+{\left[f_{4}\;\delta \left(-w^{\prime \prime }\right)\right]}_{x_{1}=0}^{x_{1}=L}-\overset{L}{\underset{0}{\int }}f_{1}^{\prime }{\;\delta U\;\mathrm{dx}}_{1}-\overset{L}{\underset{0}{\int }}f_{3}^{\prime \prime }{\;\delta w\;\mathrm{dx}}_{1}$$

The parts $T^{\left(\mathrm{cl}\right)}$ and ${\bar{T}}^{\left(\mathrm{noncl}\right)}$, which make up the total kinetic energy $\bar{T}$ in Equation (39), are defined in Equations (2) and (34), and can be calculated with the aid of Equation (56):

(72) $${Τ}^{\left(\mathrm{cl}\right)}=\overset{\;}{\underset{V}{\int }}\frac{1}{2}{\rho \dot{u}}_{k}{\dot{u}}_{k}\mathrm{dV}=\overset{L}{\underset{0}{\int }}\frac{1}{2}\rho \left[{A\dot{U}}^{2}+\;I{\left({\dot{w}}^{\prime }\right)}^{2}+A\;{\dot{w}}^{2}\;\right]\;{\mathrm{dx}}_{1},$$

(73) $${\bar{T}}^{\left(\mathrm{noncl}\right)}=\overset{\;}{\underset{V}{\int }}\frac{1}{2}\gamma \left(\partial_{j}{\dot{u}}_{k}\right)\left(\partial_{j}{\dot{u}}_{k}\right)\mathrm{dV}=\overset{L}{\underset{0}{\int }}\frac{1}{2}\gamma \left[A\;{\left({\dot{U}}^{\prime }\right)}^{2}+I{\left({\dot{w}}^{\prime \prime }\right)}^{2}+2A\;{\left({\dot{w}}^{\prime }\right)}^{2}\;\right]{\mathrm{dx}}_{1}.$$

After repeated use of partial integration and the divergence theorem, we arrive at

(74) $$\overset{t_{2}}{\underset{t_{1}}{\int }}\delta \bar{T}\mathrm{dt}\;=\overset{t_{2}}{\underset{t_{1}}{\int }}\left({\delta T}^{\left(\mathrm{cl}\right)}+{\delta \bar{T}}^{\left(\mathrm{noncl}\right)}\right)\;\mathrm{dt}\;\;=\overset{t_{2}}{\underset{t_{1}}{\int }}\{-{\left[\gamma A{\ddot{U}}^{\prime }\delta U\right]}_{x_{1}=0}^{x_{1}=L}-{\left[\left\{\left(\rho I+2\gamma A\right){\ddot{w}}^{\prime }-\gamma I{\ddot{w}}^{\prime \prime \prime }\right\}\delta w\right]}_{x_{1}=0}^{x_{1}=L}+{\left[\gamma I{\ddot{w}}^{\prime \prime }\delta \left(-w^{\prime }\right)\right]}_{x_{1}=0}^{x_{1}=L}\;\;+\overset{L}{\underset{0}{\int }}\left[\left(-\rho A\ddot{U}+\gamma A{\ddot{U}}^{\prime \prime }\right)\delta U+\left(-\rho A\ddot{w}+\left(\rho I+2\gamma A\right){\ddot{w}}^{\prime \prime }-\gamma I{\ddot{w}}^{⁗}\right)\delta w\right]{\mathrm{dx}}_{1}\}\mathrm{dt}.$$

The next step is to substitute formulas (68), (71) and (74) into Hamilton’s principle (40) and to apply the fundamental lemmas of calculus of variations. This gives the sectional constitutive laws

(75) $$\bar{N}=f_{1}+\gamma A{\ddot{U}}^{\prime }=\mathrm{EA}\;\left(U^{\prime }-l^{2}U^{\prime \prime \prime }\right)+\gamma A{\ddot{U}}^{\prime },$$

(76) $$\bar{H}=f_{2}=l^{2}\mathrm{EA}U^{\prime \prime },$$

(77) $$\bar{M}=f_{3}-\gamma I{\ddot{w}}^{\prime \prime }=-\left(\mathrm{EI}\;+l^{2}\mathrm{EA}\right)w^{\prime \prime }+l^{2}\mathrm{EIw}⁗-\gamma I{\ddot{w}}^{\prime \prime },$$

(78) $$\bar{m}=f_{4}=-l^{2}\mathrm{EI}w^{\prime \prime \prime },$$

the sectional balance law for $\bar{V}$

(79) $$\bar{V}={\left(f_{3}-\gamma Ι{\ddot{w}}^{\prime \prime }\right)}^{\prime }+\left(\rho Ι+2\gamma A\right){\ddot{w}}^{\prime }\;=-\left(\mathrm{EI}+l^{2}\mathrm{EA}\right)w^{\prime \prime \prime }+l^{2}{\mathrm{EIw}}^{\prime \prime \prime \prime \prime }-\gamma I{\ddot{w}}^{\prime \prime \prime }+\left(\rho I+2\gamma A\right){\ddot{w}}^{\prime }\;\Leftrightarrow$$

(80) $$\bar{V}-{\bar{M}}^{\prime }=\left(\rho I+2\gamma A\right){\ddot{w}}^{\prime },$$

the governing equations of motion for the axial displacement U

(81) $$\begin{array}{c} \bar{p}+f_{1}^{\prime }-\rho A\ddot{U}+\gamma A{\ddot{U}}^{\prime \prime }=0\;\Leftrightarrow \end{array}$$

(82) $$\begin{array}{c} -\rho A\ddot{U}+\gamma A{\ddot{U}}^{\prime \prime }+EAU^{\prime \prime }-l^{2}EAU^{⁗}+\bar{p}=0, \end{array}$$

the governing equation of motion for the deflection w

(83) $$-\rho A\ddot{w}+\left(\rho I+2\gamma A\right){\ddot{w}}^{\prime \prime }-\gamma I{\ddot{w}}^{⁗}+f_{3}^{\prime \prime }+\bar{q}=0\Leftrightarrow$$

(84) $$-\rho A\ddot{w}+\left(\rho I+2\gamma A\right){\ddot{w}}^{\prime \prime }-\gamma I{\ddot{w}}^{⁗}-\left(\mathrm{EI}+l^{2}\mathrm{EA}\right)w^{⁗}+l^{2}{\mathrm{EIw}}^{\prime \prime \prime \prime \prime \prime }+\bar{q}=0$$

and the boundary conditions

(85) $$\mathrm{either}\bar{N}\mathrm{or}\;U,\;\mathrm{either}\bar{H}\mathrm{or}U^{\prime },\;\mathrm{either}\bar{V}\mathrm{or}\;w,$$

(86) $$\mathrm{either}\bar{M}\mathrm{or}w^{\prime }\mathrm{and}\;\mathrm{either}\bar{m}\mathrm{or}w^{\prime \prime }$$

have to be prescribed at $x_{1}=0{\;\mathrm{and}\;x}_{1}=L$.

Now, it will be shown that the variational formulation (40), with ${\delta \bar{W}}^{\left(e\right)}$, $\Pi^{\;\left(i\right)}$ and $\bar{T}=T^{\left(\mathrm{cl}\right)}+{\bar{T}}^{\left(\mathrm{noncl}\right)}$ being given in Equations (68), (69), (72) and (73), respectively, can be converted into an equivalent reduced form.

##### **Approach Based on the Balance of Linear Momentum for the Beam** 

Alternatively, one might establish a formulation of Hamilton’s principle on the basis of the balance equations of linear momentum for the beam in (63) and (64), which can be rewritten with the help of the stress components $\Sigma_{\mathrm{ij}}^{\left(t\right)}$ defined in Equation (37):

(87) $$\partial_{1}\Sigma_{11}^{\left(t\right)}+\partial_{3}\Sigma_{31}^{\left(t\right)}=\rho {\ddot{u}}_{1},$$

(88) $$\partial_{1}\Sigma_{13}^{\left(t\right)}+\partial_{3}\Sigma_{33}^{\left(t\right)}=\rho {\ddot{u}}_{3},$$

with

(89) $$\Sigma_{11}^{\left(t\right)}=\Sigma_{11}+\gamma \left({\ddot{U}}^{\prime }-{\ddot{w}}^{\prime \prime }x_{3}\right),{\;\Sigma }_{31}^{\left(t\right)}=\Sigma_{31}-\gamma {\ddot{w}}^{\prime },$$

(90) $$\Sigma_{13}^{\left(t\right)}=\Sigma_{13}+\gamma {\ddot{w}}^{\prime }\;,{\;\Sigma }_{33}^{\left(t\right)}=\Sigma_{33}.$$

Scalar multiplication of Equations (87) and (88) with the virtual displacement vector δ**u** (see Equation (56)), and integration over V, yields

(91) $$\overset{\;}{\underset{V}{\int }}\left[\left(\partial_{1}\Sigma_{11}^{\left(t\right)}+\partial_{3}\Sigma_{31}^{\left(t\right)}\right){\delta u}_{1}\right]+\left[\left(\partial_{1}\Sigma_{13}^{\left(t\right)}+\partial_{3}\Sigma_{33}^{\left(t\right)}\right){\delta u}_{3}\right]\mathrm{dV}\;-\overset{\;}{\underset{V}{\int }}\rho \left(\rho {\ddot{u}}_{1}{\delta u}_{1}+\rho {\ddot{u}}_{3}{\delta u}_{3}\right)\mathrm{dV}=0.$$

In order to replace the stress components by sectional and resultant forces, we introduce the following definitions

(92) $${\bar{N}}_{r}:=\overset{\;}{\underset{A}{\int }}\Sigma_{11}^{\left(t\right)}\mathrm{dS}\;,{\bar{H}}_{r}\;:=\overset{\;}{\underset{A}{\int }}\mu_{111}\mathrm{dS},{\bar{V}}_{r}:=-\overset{\;}{\underset{A}{\int }}\left(\partial_{3}\Sigma_{31}^{\left(t\right)}\right)\;\mathrm{dS}\;,$$

(93) $${\bar{M}}_{r}:=\overset{\;}{\underset{A}{\int }}\Sigma_{11}^{\left(t\right)}x_{3}\mathrm{dS}\;,{\bar{m}}_{r}\;:=\overset{\;}{\underset{A}{\int }}\mu_{111}x_{3}\mathrm{dS},$$

(94) $${\bar{p}}_{r}:=2b{\left[\Sigma_{31}^{\left(t\right)}\right]}_{x_{3}=-c}^{x_{3}=c},{\bar{q}}_{r}:=2b{\left[\Sigma_{33}+\left(\partial_{1}\Sigma_{13}\right)x_{3}\right]}_{x_{3}=-c}^{x_{3}=c}+\gamma A{\ddot{w}}^{\prime \prime }.$$

Using these forces, it is shown in Appendix A that Equation (91) is equivalent to the following virtual work statement for the beam, which is of the form (12):

(95) $${\delta \bar{W}}_{r}^{\left(\mathrm{inert}\right)}+{\delta \bar{W}}_{r}^{\left(e\right)}-{\delta \Pi }_{r}^{\left(i\right)}=0,$$

with

(96) $${\delta \bar{W}}_{r}^{\left(\mathrm{inert}\right)}:=\overset{L}{\underset{0}{\int }}\left(-\rho A\ddot{U}\delta U+\rho I{\ddot{w}}^{\prime }\delta w^{\prime }+\rho A\ddot{w}\delta w\right){\mathrm{dx}}_{1}\;-\overset{L}{\underset{0}{\int }}\left(\gamma A{\ddot{U}}^{\prime }\delta U^{\prime }+\gamma I{\ddot{w}}^{\prime \prime }\delta w^{\prime \prime }\right){\mathrm{dx}}_{1},$$

(97) $${\delta \bar{W}}_{r}^{\left(e\right)}\;:={\left[{\bar{N}}_{r}\;\delta U\right]}_{x_{1}=0}^{x_{1}=L}+{\left[{\bar{H}}_{r}\delta U^{\prime }\right]}_{x_{1}=0}^{x_{1}=L}+{\left[{\bar{V}}_{r}\delta w\right]}_{x_{1}=0}^{x_{1}=L}+{\left[{\bar{M}}_{r}\;\delta \left(-w^{\prime }\right)\right]}_{x_{1}=0}^{x_{1}=L}\;+{\left[{\bar{m}}_{r}\;\delta \left(-w^{\prime \prime }\right)\right]}_{x_{1}=0}^{x_{1}=L}+\overset{L}{\underset{0}{\int }}{\bar{p}}_{r}{\;\delta U\;\mathrm{dx}}_{1}+\overset{L}{\underset{0}{\int }}{\bar{q}}_{r}{\;\delta w\;\mathrm{dx}}_{1},$$

(98) $$\Pi_{r}^{\left(i\right)}:=\overset{\;}{\underset{V}{\int }}\frac{1}{2}E\left[\varepsilon_{11}^{2}+l^{2}{\left(\partial_{1}\varepsilon_{11}\right)}^{2}\right]\mathrm{dV}.$$

It is readily seen, by using partial integration, that the time integral of ${\delta \bar{W}}_{r}^{\left(\mathrm{inert}\right)}$ between $t_{1}$ and $t_{2}$ can be represented in terms of kinetic energy ${\bar{T}}_{r}$,

(99) $${\bar{T}}_{r}:=T^{\left(\mathrm{cl}\right)}+\overset{L}{\underset{0}{\int }}\frac{1}{2}\gamma \left[A{\left({\dot{U}}^{\prime }\right)}^{2}-I{\left({\dot{w}}^{\prime \prime }\right)}^{2}\right]{\;\mathrm{dx}}_{1}$$

with $T^{\left(\mathrm{cl}\right)}$ as given in Equation (72), i.e.,

(100) $$\overset{t_{2}}{\underset{t_{1}}{\int }}{\delta \bar{W}}_{r}^{\left(\mathrm{inert}\right)}\;\mathrm{dt}\;=\;\delta \overset{t_{2}}{\underset{t_{1}}{\int }}{\bar{T}}_{r}\;\mathrm{dt}.$$

Thus, by taking the time integral of (95), we arrive at the variational formulation

(101) $$\delta \overset{t_{2}}{\underset{t_{1}}{\int }}\left({\bar{T}}_{r}-\Pi_{r}^{\left(i\right)}\right)\;\mathrm{dt}\;+\overset{t_{2}}{\underset{t_{1}}{\int }}{\delta \bar{W}}_{r}^{\left(e\right)}\;\mathrm{dt}=0,$$

which is of the form (10).

We would like to draw attention to the fact that $\Pi_{r}^{\left(i\right)}$ in Equation (98) includes one term less than $\Pi^{\left(i\right)}$ in Equation (66). That is why we consider (101) as a reduced form of the principle (40).

Now, using partial integration repeatedly, the variation of $\Pi_{r}^{\left(i\right)}$ may be expressed in terms of displacement components as follows:

(102) $${\delta \Pi }_{r}^{\left(i\right)}={\left[f_{1}\delta U\right]}_{x_{1}=0}^{x_{1}=L}+{\left[f_{2}\delta U^{\prime }\right]}_{x_{1}=0}^{x_{1}=L}+{\left[\left(f_{3}^{\prime }+l^{2}\mathrm{EA}w^{\prime \prime \prime }\right)\delta w\right]}_{x_{1}=0}^{x_{1}=L}\;+{\left[\left(f_{3}+l^{2}\mathrm{EA}w^{\prime \prime }\right)\delta \left(-w^{\prime }\right)\right]}_{x_{1}=0}^{x_{1}=L}+{\left[f_{4}\;\delta \left(-w^{\prime \prime }\right)\right]}_{x_{1}=0}^{x_{1}=L}\;-\overset{L}{\underset{0}{\int }}f_{1}^{\prime }{\;\delta U\;\mathrm{dx}}_{1}-\overset{L}{\underset{0}{\int }}\left(f_{3}^{\prime \prime }+l^{2}{\mathrm{EAw}}^{⁗}\right){\delta \mathrm{wdx}}_{1}.$$

Similarly, from Equation (96) we obtain

(103) $${\delta \bar{W}}_{r}^{\left(\mathrm{inert}\right)}:=-{\left[\gamma A{\ddot{U}}^{\prime }\delta U\right]}_{x_{1}=0}^{x_{1}=L}-{\left[\left(\rho I{\ddot{w}}^{\prime }-\gamma I{\ddot{w}}^{\prime \prime \prime }\right)\delta w\right]}_{x_{1}=0}^{x_{1}=L}-{\left[\gamma I{\ddot{w}}^{\prime \prime }\;\delta w^{\prime }\right]}_{x_{1}=0}^{x_{1}=L}\;+\overset{L}{\underset{0}{\int }}\left(-\rho A\ddot{U}+\gamma A{\ddot{U}}^{\prime \prime }\right){\delta \mathrm{Udx}}_{1}+\overset{L}{\underset{0}{\int }}\left(-\rho A\ddot{w}+\rho I{\ddot{w}}^{\prime \prime }+\gamma I{\ddot{w}}^{⁗}\right){\delta w\;\mathrm{dx}}_{1}.$$

Substitution of (97), (102) and (103) into (95) and application of the fundamental lemmas of calculus of variations leads to the sectional constitutive laws

(104) $${\bar{N}}_{r}=f_{1}+\gamma A{\ddot{U}}^{\prime }=\mathrm{EA}\left(U^{\prime }-l^{2}U^{\prime \prime \prime }\right)+\gamma A{\ddot{U}}^{\prime },$$

(105) $${\bar{H}}_{r}=f_{2}=l^{2}\mathrm{EA}U^{\prime \prime },$$

(106) $${\bar{M}}_{r}=f_{3}+l^{2}\mathrm{EA}w^{\prime \prime }-\gamma I{\ddot{w}}^{\prime \prime }=-\mathrm{EI}\left(w^{\prime \prime }-l^{2}w^{⁗}\right)-\gamma I{\ddot{w}}^{\prime \prime }\;,$$

(107) $${\bar{m}}_{r}=f_{4}=-l^{2}\mathrm{EI}w^{\prime \prime \prime },$$

the sectional balance law for ${\bar{V}}_{r}$

(108) $${\bar{V}}_{r}=f_{3}^{\prime }+l^{2}\mathrm{EA}w^{\prime \prime \prime }+\rho I{\ddot{w}}^{\prime }-\gamma I{\ddot{w}}^{\prime \prime \prime }\;=-\mathrm{EI}w^{\prime \prime \prime }+l^{2}{\mathrm{EIw}}^{\prime \prime \prime \prime \prime }+\rho I{\ddot{w}}^{\prime }-\gamma I{\ddot{w}}^{\prime \prime \prime }\Leftrightarrow$$

(109) $${\bar{V}}_{r}-{\bar{M}}_{r}^{\prime }=\rho I{\ddot{w}}^{\prime },$$

the governing equation of motion for the axial displacement U

(110) $${\bar{p}}_{r}+f_{1}^{\prime }-\rho A\ddot{U}+\gamma A{\ddot{U}}^{\prime \prime }=0\;\Leftrightarrow$$

(111) $$-\rho A\ddot{U}+\gamma A{\ddot{U}}^{\prime \prime }+\mathrm{EA}U^{\prime \prime }-l^{2}{\mathrm{EAU}}^{⁗}+{\bar{p}}_{r}=0,$$

the governing equation of motion for the deflection w

(112) $${\bar{q}}_{r}+f_{3}^{\prime \prime }+l^{2}{\mathrm{EAw}}^{⁗}-\rho A\ddot{w}+\rho I{\ddot{w}}^{\prime \prime }-\gamma I{\ddot{w}}^{⁗}=0\Leftrightarrow$$

(113) $$-\rho A\ddot{w}+\rho I{\ddot{w}}^{\prime \prime }-\gamma I{\ddot{w}}^{⁗}-{\mathrm{EIw}}^{⁗}+l^{2}{\mathrm{EIw}}^{\prime \prime \prime \prime \prime \prime }+{\bar{q}}_{r}=0$$

and the boundary conditions

(114) $$\mathrm{either}\;{\bar{N}}_{r}\;\mathrm{or}\;U,{\;\mathrm{either}\bar{H}}_{r}\;\mathrm{or}U^{\prime },{\;\mathrm{either}\bar{V}}_{r}\;\mathrm{or}\;w,$$

(115) $$\mathrm{either}\;{\bar{M}}_{r\;}{\mathrm{or}w^{\prime }\mathrm{and}\;\mathrm{either}\bar{m}}_{r}\;\mathrm{or}w^{\prime \prime }$$

have to be prescribed at $x_{1}=0$ and $x_{1}=L$.

It should be noted that the sectional constitutive laws (104)–(107) can alternatively be derived from definitions (92) and (93). Also, a bending theory based on $\Pi_{r}^{\left(i\right)}$ in Equation (98) has been formulated for the first time in Papargyri-Beskou et al. [27].

The two beam approaches considered in this section are equivalent to each other. To see this, it suffices to show that the second approach implies the first one. Let $p_{0}$ be a given loading function and assume that $\bar{p}={\bar{p}}_{r}=p_{0}$. Then, the two equations of motion (82) and (111) are identical. Further, assume ${\bar{q}}_{r}$ to have the form ${\bar{q}}_{r}=q_{0}-l^{2}{\mathrm{EAw}}^{⁗}+2\gamma A{\ddot{w}}^{\prime \prime }$ with $q_{0}$ being a known external body force. Then, the equation of motion (113) implies the equation of motion (84) with $\bar{q}=q_{0}$. The displacement boundary conditions and the traction boundary conditions for $\bar{N}$, $\bar{H}$, $\bar{m,}$ and ${\bar{N}}_{r}$, ${\bar{H}}_{r}$, ${\bar{m}}_{r}$ are identical for both approaches. By comparing Equation (79) with (108) and Equation (77) with (106), we recognize that $\bar{V}={\bar{V}}_{r}-l^{2}\mathrm{EA}w^{\prime \prime \prime }+2\gamma A{\ddot{w}}^{\prime }$ and $\bar{M}={\bar{M}}_{r}-l^{2}\mathrm{EA}w^{\prime \prime }$. Thus, assume that at the boundaries $x_{1}=0,\;L$, ${\bar{V}}_{r}$ and ${\bar{M}}_{r}$ have the forms ${\bar{V}}_{r}=V_{0}+l^{2}\mathrm{EA}w^{\prime \prime \prime }-2\gamma A{\ddot{w}}^{\prime }$, ${\bar{M}}_{r}=M_{0}+l^{2}\mathrm{EA}w^{\prime \prime },{\;\mathrm{with}\;V}_{0}$, $M_{0}$ being given. Then, to the boundary conditions for ${\bar{V}}_{r}$ and ${\bar{M}}_{r}$ correspond the boundary conditions $\bar{V}=V_{0}$ and $\bar{M}=M_{0}$. Note that such boundary conditions include acceleration terms, but this is characteristic of Mindlin’s approach. Finally, by adding the time integral of

(116) $$\delta \overset{\;}{\underset{V}{\int }}\frac{1}{2}l^{2}E{\left(\partial_{3}\varepsilon_{11}\right)}^{2}\mathrm{dV}+{\left[l^{2}\mathrm{EA}w^{\prime \prime \prime }\delta w\right]}_{x_{1}=0}^{x_{1}=L}+{\left[l^{2}\mathrm{EA}w^{\prime \prime }\delta \left(-w^{\prime }\right)\right]}_{x_{1}=0}^{x_{1}=L}\;-\overset{L}{\underset{0}{\int }}l^{2}{\mathrm{EAw}}^{⁗}{\delta w\;\mathrm{dx}}_{1}=0\;$$

and the identity

(117) $$-\delta \overset{t_{2}}{\underset{t_{1}}{\int }}\overset{L}{\underset{0}{\int }}\gamma A{\left({\dot{w}}^{\prime }\right)}^{2}{\mathrm{dx}}_{1}\mathrm{dt}+\overset{t_{2}}{\underset{t_{1}}{\int }}\overset{L}{\underset{0}{\int }}2\gamma A{\ddot{w}}^{\prime \prime }{\delta \mathrm{wdx}}_{1}\mathrm{dt}-\delta \overset{t_{2}}{\underset{t_{1}}{\int }}{\left[2\gamma A{\ddot{w}}^{\prime }\delta w\right]}_{x_{1}=0}^{x_{1}=L}\mathrm{dt}=0$$

to Hamilton’s principle (101), and replacing ${\bar{V}}_{r}-l^{2}\mathrm{EA}w^{\prime \prime \prime }+2\gamma A{\ddot{w}}^{\prime }$ by $\bar{V}$ and ${\bar{M}}_{r}-l^{2}\mathrm{EA}w^{\prime \prime }$ by $\bar{M}$, we can deduce that (101) implies Hamilton’s principle (40) with ${\delta \bar{W}}^{\left(e\right)}$, $\Pi^{\;\left(i\right)}$ and $\bar{T}=T^{\left(\mathrm{cl}\right)}+{\bar{T}}^{\left(\mathrm{noncl}\right)}$ as given in Equation (68), (70), (72) and (73), respectively.

#### 4.2.2. Hamilton’s Principle for the Case Where Acceleration Terms Are Not Present in the Traction Boundary Conditions

Similar to Section 4.2.1, again, there are two equivalent methods for deriving the equations of motion. The first one starts with Hamilton’s principle (48), assuming ${\delta \hat{W}}^{\left(e\right)}$ and ${\delta \hat{W}}^{\left(\mathrm{inert},\;\mathrm{noncl}\right)}$ are as defined in Equations (49) and (47), respectively. The main feature of this method is that it deals with the potential for the internal forces $\Pi^{\;\left(i\right)}$ in Equation (69). The second one starts with the equations of linear momentum (63) and (64) and leads to the reduced form of the potential of internal forces $\Pi_{r}^{\left(i\right)}$ in Equation (98). The calculations are quite similar to those in Section 4.2.1. In the examples below, we shall employ only the equations of motion derived according to the second method. Therefore, only this method will be discussed here. Accordingly, some quantities are designated with the subscript r.

In the last section, the balance of linear momentum of the beam (Equations (87) and (88)), expressed in terms of the total Cauchy stress tensor $\Sigma^{\left(t\right)}$, was the start point. Opposite to this, we now start with the balance of linear momentum in Equations (63) and (64), which is expressed in terms of the proper Cauchy stress **Σ**:

(118) $$\partial \Sigma_{11}+\partial_{3}\Sigma_{13}+i_{1}=0,$$

(119) $$\partial \Sigma_{13}+\partial_{3}\Sigma_{33}+i_{3}=0,$$

with the components of the inertial force **i** being given by

(120) $$i_{1}=i_{1}^{\left(\mathrm{cl}\right)}+i_{1}^{\left(\mathrm{noncl}\right)},{\;i}_{1}^{\left(\mathrm{cl}\right)}=-\rho {\ddot{u}}_{1},{\;i}_{1}^{\left(\mathrm{noncl}\right)}=\gamma {\ddot{u}}_{1}^{\prime \prime },$$

(121) $$i_{3}=i_{3}^{\left(\mathrm{cl}\right)}+i_{3}^{\left(\mathrm{noncl}\right)},{\;i}_{3}^{\left(\mathrm{cl}\right)}=-\rho {\ddot{u}}_{3},{\;i}_{1}^{\left(\mathrm{noncl}\right)}=\gamma {\ddot{u}}_{3}^{\prime \prime }.$$

By scalar multiplication of (118) and (119) with virtual displacement (see Equation (56)) and integration over V, we find that

(122) $$\overset{\;}{\underset{V}{\int }}\left[\left(\partial \Sigma_{11}+\partial_{3}\Sigma_{13}\right){\delta u}_{1}+\left(\partial \Sigma_{13}+\partial_{3}\Sigma_{33}\right){\delta u}_{3}\right]\mathrm{dV}+{\delta W}_{r}^{\left(\mathrm{inert}\right)}=0.$$

In this equation, ${\delta W}_{r}^{\left(\mathrm{inert}\right)}$ is the virtual work of the inertial force composed of a classical and a non-classical term,

(123) $${\delta W}_{r}^{\left(\mathrm{inert}\right)}={\delta W}^{\left(\mathrm{inert},\mathrm{cl}\right)}+{\delta W}_{r}^{\left(\mathrm{inert},\mathrm{noncl}\right)},$$

(124) $${\delta W}_{r}^{\left(\mathrm{inert},\mathrm{cl}\right)}=\overset{\;}{\underset{V}{\int }}i_{i}^{\left(\mathrm{cl}\right)}{\delta u}_{i}\mathrm{dV}=-\overset{\;}{\underset{V}{\int }}\left(\rho {\ddot{u}}_{1}{\delta u}_{1}+\rho {\ddot{u}}_{3}{\delta u}_{3}\right)\mathrm{dV}\;=-\overset{L}{\underset{0}{\int }}(\rho A\ddot{U}\delta U+\rho I{\ddot{w}}^{\prime }\delta w^{\prime }+\rho A\ddot{w}\delta w){\;\mathrm{dx}}_{1}\;=-{\left[\rho I{\ddot{w}}^{\prime }\delta w\right]}_{x_{1}=0}^{x_{1}=L}+\overset{L}{\underset{0}{\int }}[-\rho A\ddot{U}\delta U-\left(\rho A\ddot{w}-\rho I{\ddot{w}}^{\prime \prime }\right)\delta w]{\;\mathrm{dx}}_{1},$$

(125) $${\delta W}_{r}^{\left(\mathrm{inert},\mathrm{noncl}\right)}=\overset{\;}{\underset{V}{\int }}i_{i}^{\left(\mathrm{noncl}\right)}{\delta u}_{i}\mathrm{dV}=\overset{\;}{\underset{V}{\int }}\left(\gamma {\ddot{u}}_{1}^{\prime \prime }{\delta u}_{1}+\gamma {\ddot{u}}_{3}^{\prime \prime }{\delta u}_{3}\right)\mathrm{dV}\;=\overset{L}{\underset{0}{\int }}(\gamma A{\ddot{U}}^{\prime \prime }\delta U+\gamma I{\ddot{w}}^{\prime \prime \prime }\delta w^{\prime }){\;\mathrm{dx}}_{1}\;={\left[\gamma I{\ddot{w}}^{\prime \prime \prime }\delta w\right]}_{x_{1}=0}^{x_{1}=L}+\overset{L}{\underset{0}{\int }}\left(\gamma A{\ddot{U}}^{\prime \prime }\delta U-\gamma I{\ddot{w}}^{⁗}\delta w\right){\mathrm{dx}}_{1}.$$

The appropriate definitions of sectional and resultant forces are now

(126) $$N_{r}:=\overset{\;}{\underset{A}{\int }}\Sigma_{11}\mathrm{dS},{\;H}_{r}:=\overset{\;}{\underset{A}{\int }}\mu_{111}\mathrm{dS},{\;V}_{r}:=-\overset{\;}{\underset{A}{\int }}\left(\partial_{3}\Sigma_{13}\right)x_{3}\mathrm{dS},$$

(127) $$M_{r}:=\overset{\;}{\underset{A}{\int }}\Sigma_{11}x_{3}\mathrm{dS},{\;m}_{r}:=\overset{\;}{\underset{A}{\int }}\mu_{111}x_{3}\mathrm{dS},$$

(128) $$p_{r}:=2b\;{\left[\Sigma_{13}\right]}_{x_{3}=-c}^{x_{3}=c}\;,{\;q}_{r}:=2b\;{\left[\Sigma_{33}+\left(\partial_{1}\Sigma_{13}\right)x_{3}\right]}_{x_{3}=-c}^{x_{3}=c}.$$

It is worth noting that these definitions apply equally in statics (cf. [25]). With the help of these definitions, it can be shown (see Appendix B), that Equation (122) implies

(129) $${\delta W}_{r}^{\left(\mathrm{inert}\right)}+{\delta W}_{r}^{\left(e\right)}-{\delta \Pi }_{r}^{\left(i\right)}=0,$$

where

(130) $${\delta W}_{r}^{\left(e\right)}={\left[N_{r}\;\delta U\right]}_{x_{1}=0}^{x_{1}=L}+{\left[H_{r}\delta U^{\prime }\right]}_{x_{1}=0}^{x_{1}=L}+{\left[V_{r}\delta w\right]}_{x_{1}=0}^{x_{1}=L}+{\left[M_{r}\delta \left(-w^{\prime }\right)\right]}_{x_{1}=0}^{x_{1}=L}\;+{\left[m_{r}\delta \left(-w^{\prime \prime }\right)\right]}_{x_{1}=0}^{x_{1}=L}+\overset{L}{\underset{0}{\int }}p_{r}{\delta \mathrm{Udx}}_{1}+\overset{L}{\underset{0}{\int }}q_{r}{\delta \mathrm{wdx}}_{1}.$$

Equation (129) is a virtual work principle of the form (12). We recall the well-known identity (cf. Equation (125))

(131) $$\overset{t_{2}}{\underset{t_{1}}{\int }}{\delta W}^{\left(\mathrm{inert},\mathrm{cl}\right)}\mathrm{dt}=\delta \overset{t_{2}}{\underset{t_{1}}{\int }}\frac{1}{2}{\left[{\rho A\dot{U}}^{2}+\rho I({\dot{w}}^{\prime }\right)}^{2}+{\rho A\dot{w}}^{2}]\mathrm{dt}=\delta \overset{t_{2}}{\underset{t_{1}}{\int }}T^{\left(\mathrm{cl}\right)}\mathrm{dt},$$

and that Equation (123) applies. Thus, time integration of Equation (129) furnishes

(132) $$\delta \overset{t_{2}}{\underset{t_{1}}{\int }}\left(T^{\left(\mathrm{cl}\right)}-\Pi^{\;\left(i\right)}\right)\mathrm{dt}+\overset{t_{2}}{\underset{t_{1}}{\int }}\left({\delta W}_{r}^{\left(e\right)}+{\delta W}_{r}^{\left(\mathrm{inert},\mathrm{noncl}\right)}\right)\mathrm{dt}=0,$$

which is a variant of Hamilton’s principle of the form (11).

On the other hand, we can invoke in Equation (129) the result (102) for ${\delta \Pi }_{r}^{\left(i\right)}$ and the result (123), (124)_3_ and (125)_3_ for ${\delta W}_{r}^{\left(\mathrm{inert}\right)}$, and by applying the fundamental lemmas of calculus for variations to infer the sectional constitutive laws

(133) $$N_{r}=f_{1}=\mathrm{EA}\left(U^{\prime }-l^{2}U^{\prime \prime \prime }\right),$$

(134) $$H_{r}=f_{2}=l^{2}\mathrm{EA}U^{\prime \prime },$$

(135) $$M_{r}=f_{3}+l^{2}\mathrm{EA}w^{\prime \prime }=-\mathrm{EI}\left(w^{\prime \prime }-l^{2}w^{⁗}\right),$$

(136) $$m_{r}=f_{4}=-l^{2}\mathrm{EI}w^{\prime \prime \prime },$$

the sectional balance law for $V_{r}$

(137) $$V_{r}=f_{3}^{\prime }+l^{2}\mathrm{EA}w^{\prime \prime \prime }+\rho I{\ddot{w}}^{\prime \prime }-\gamma I{\ddot{w}}^{\prime \prime \prime }\;=-ΕΙw^{\prime \prime \prime }+l^{2}{\mathrm{EI}\;w}^{\prime \prime \prime \prime \prime }+\rho I{\ddot{w}}^{\prime }-\gamma I{\ddot{w}}^{\prime \prime \prime }\;\Leftrightarrow$$

(138) $$V_{r}-M_{r}^{\prime }=\rho I{\ddot{w}}^{\prime }-\gamma I{\ddot{w}}^{\prime \prime \prime },$$

the governing equation of motion for the axial displacement U

(139) $$p_{r}+f_{1}^{\prime }-\rho A\ddot{U}+\gamma A{\ddot{U}}^{\prime \prime }\Leftrightarrow$$

(140) $$-\rho A\ddot{U}+\;\gamma A{\ddot{U}}^{\prime \prime }+\mathrm{EA}U^{\prime \prime }-l^{2}{\mathrm{EAU}}^{\prime \prime \prime \prime \prime }+p_{r}=0,$$

the governing equation of motion for the deflection w

(141) $$q_{r}+f_{3}^{\prime \prime }+l^{2}{\mathrm{EA}\;w}^{⁗}-\rho A\ddot{w}+\rho I{\ddot{w}}^{\prime \prime }-\gamma I{\ddot{w}}^{⁗}=0\Leftrightarrow$$

(142) $$-\rho A\ddot{w}+\rho I{\ddot{w}}^{\prime \prime }-\gamma I{\ddot{w}}^{⁗}-{\mathrm{EIw}}^{⁗}+l^{2}{\mathrm{EI}\;w}^{\prime \prime \prime \prime \prime \prime }+q_{r}=0\;$$

and the boundary conditions

(143) $${\mathrm{either}\;N}_{r}\;\mathrm{or}\;U,{\;\mathrm{either}\;H}_{r}\;\mathrm{or}U^{\prime },{\;\mathrm{either}\;V}_{r}\;\mathrm{or}\;w,$$

(144) $${\mathrm{either}\;M}_{r}{\;\mathrm{or}\;w^{\prime }\mathrm{and}\;\mathrm{either}\;m}_{r}\;\mathrm{or}\;w^{\prime \prime }$$

have to be prescribed at $x_{1}=0\;$ and $x_{1}=L\;$.

We see from Equations (104)–(113) and (133)–(142), that differences between the two approaches only exist in the boundary conditions for the sectional forces; some contain acceleration terms while others do not.

## 5. Examples

The proper aim of the examples is to demonstrate with reference to bending loading that the presence of acceleration terms in the boundary conditions may have significant qualitative and quantitative effect on the predicted responses. Note, however, that Euler–Bernoulli beam theories are especially tempting for they render the equations of motion one-dimensional. That naturally arouses interest to compare with each other predicted responses according to one-dimensional tension/compression and bending loadings. For our purposes, it suffices to perform the comparison only with respect to the beam approaches, which rely upon the reduced form of the potential of internal forces. Additionally, we shall concentrate on the following three versions of the theory.

Version 1: γ = 0, i.e., nonclassical acceleration terms are omitted.

Version 2: γ≠0, nonclassical acceleration terms are present in both the equations of motion and the traction boundary conditions.

Version 3: γ≠0, nonclassical acceleration terms are present only in the equations of motion.

In the case of Version 1, the equations of motion follow from (140) and (142) by setting γ = 0, while the boundary conditions are the same as for Version 3. Furthermore, the definitions of sectional and resultant distributed forces for Version 1 are the same as for Version 3. The predicted responses will be presented in dimensionless form by employing the definitions

(145) x˜:=x1L ,t˜:=c*L t ,∂ ∂x˜= , x˜ , ∂ ∂t˜= , t˜ ,U˜:=UL ,w˜:=wL ,N˜:=NEA,

(146) $$\tilde{V}:=\frac{V}{{\mathrm{EL}}^{2}}\;,\tilde{M}:=\frac{M}{{\mathrm{EL}}^{3}}\;,\tilde{\gamma }:=\frac{{\gamma c}^{*2}}{{\mathrm{EL}}^{2}}\;,{\;c}^{*}=\sqrt{\frac{E}{\rho }}\;,\tilde{A}:=\frac{A}{L^{2}},\tilde{I}:=\frac{I}{L^{4}},\;\tilde{l}:=\frac{l}{L},\tilde{\omega }:=\frac{\omega L}{c^{*}}.$$

Here, N =${\;N}_{r}$, V =${\;V}_{r}$, M =${\;M}_{r}$ for Versions 1, 3, while N = ${\bar{N}}_{r}$, V = ${\bar{V}}_{r}\;,\;M\;={\bar{M}}_{r}$ for Version 2. We shall compare the three versions with each other for the case of a cantilever beam and for harmonically with time-varying loading conditions. In all calculations, the values of $\tilde{A}=15\;\cdot 10^{-4}$ and $\tilde{I}=2.813\;\cdot 10^{-9}$ have been chosen.

### 5.1. Uniaxial Tension/Compression Loading

#### 5.1.1. Governing Equations

Consider a cantilever beam, subject only to axial load, so that w = 0, and let ${\bar{p}}_{r}=p_{r}=0.$ Then, from Equation (111) or (140), we find that

(147) $$-\;\rho \ddot{U}+\gamma {\ddot{U}}^{\prime \prime }+EU^{\prime \prime }-l^{2}{E\;U}^{⁗}=0,$$

or in dimensionless form

(148) $$-\tilde{U}{,}_{\tilde{t}\tilde{t}}+\tilde{\gamma }\tilde{U}{,}_{\tilde{x}\tilde{x}\tilde{t}\tilde{t}}+\tilde{U}{,}_{\tilde{x}\tilde{x}}-{\tilde{l}}^{2}\tilde{U}{,}_{\tilde{x}\tilde{x}\tilde{x}\tilde{x}}=0.$$

If $\tilde{\gamma }=0,\;$ then (148) represents the equation of motion for Version 1 and if $\tilde{\gamma }\neq 0$, then (148) is the equation of motion for versions 2, 3.

A comprehensive discussion of Equation (148), with respect to size effects and the convergence behavior for $\tilde{l}\to 0$, has been provided in Broese et al. [15], but with nonclassical boundary conditions different from those we shall assume in the present paper. Especially, the interest here is focused on homogenous nonclassical traction boundary tractions. The important loading condition is at $x_{1}=L$ and has the form $B_{A}e^{i\omega t}$, with ω being an operating frequency, $B_{A}$ being a displacement-or force-like amplitude and i being the imaginary unit. This kind of loading suggests assuming for the solution of Equation (148) the form

(149) $$\tilde{U}\left(\tilde{x},\tilde{t}\right)={\tilde{U}}_{0}\left(\tilde{x}\right){\;e}^{i\tilde{\omega }\tilde{t}}\;.\;$$

After substitution of this into Equation (148), and elimination of the factor $e^{i\tilde{\omega }\tilde{t}}$, we obtain

(150) $${\tilde{\omega }\tilde{U}}_{0}+\left(1-{\tilde{\gamma }\tilde{\omega }}^{2}\right)({\tilde{U}}_{0}){,}_{\tilde{x}\tilde{x}}-{\tilde{l}}^{2}({\tilde{U}}_{0}){,}_{\tilde{x}\tilde{x}\tilde{x}\tilde{x}}=0,$$

which, along with a set of boundary conditions, can be solved by employing standard methods. Having available solution of displacement (149), it is straightforward to establish solutions of sectional forces from the corresponding formulae. We will now discuss force and displacement-controlled loadings for a cantilever beam.

#### 5.1.2. Force Controlled Loading

The boundary conditions for Versions 1, 3 are $U\left(0,\;t\right)=0,{\;H}_{r}\left(0,t\right)=H_{r}\left(L,t\right)=0$ and $N\left(L,t\right)=F_{A}e^{i\omega t}$, where $F_{A}$ = constant is a force amplitude. From these, we can gain boundary conditions for the differential Equation (150) by taking Equations (133), (134) and (149) into account, eliminating the factor $e^{i\omega t}$, and using dimensionless variables. In particular, the dimensionless expression of the boundary condition for N becomes $\tilde{N}\left(1,\tilde{t}\right)={\tilde{F}}_{A}e^{i\tilde{\omega }\tilde{t}}$, with ${\tilde{F}}_{A}=\frac{F_{A}}{\mathrm{EA}}$. Moreover, it is readily seen from Equation (133), that, for Versions 1, 3, $\tilde{N}={\tilde{N}}_{0}\left(\tilde{x}\right){\;e}^{i\tilde{\omega }\tilde{t}}$, with ${\tilde{N}}_{0}=({\tilde{U}}_{0}){,}_{\tilde{x}}-{\tilde{l}}^{2}({\tilde{U}}_{0}){,}_{\tilde{X}\tilde{X}\tilde{X}}$. With the help of Equations (104) and (105) and using similar manipulations as for Versions 1, 3, it is straightforward to establish corresponding boundary conditions and distributions of ${\tilde{N}}_{0}$ for Version 2, where again $\tilde{N}={\tilde{N}}_{0}\left(\tilde{x}\right)e^{i\tilde{\omega }\tilde{t}}$. Altogether, we have the following boundary conditions and distributions of ${\tilde{N}}_{0}$.

Versions 1, 3

$$B.C.:{\left[{\tilde{U}}_{0}\right]}_{\tilde{x}=0}=[({\tilde{U}}_{0}){,}_{\tilde{x}\tilde{x}}{]}_{\tilde{x}=0}=[({\tilde{U}}_{0}){,}_{\tilde{x}\tilde{x}}{]}_{\tilde{x}=1}=0,$$

(151) $$[({\tilde{U}}_{0}){,}_{\tilde{x}}-{\tilde{l}}^{2}({\tilde{U}}_{0}){,}_{\tilde{x}\tilde{x}\tilde{x}}{]}_{\tilde{x}=1}={\tilde{F}}_{A}.$$

(152) $${\mathrm{Solution}\;\tilde{N}}_{0}:{\tilde{N}}_{0}\left(\tilde{x}\right)=({\tilde{U}}_{0}){,}_{\tilde{x}}-{\tilde{l}}^{2}({\tilde{U}}_{0}){,}_{\tilde{x}\tilde{x}\tilde{x}}$$

Version 2

$$B.C.:{\left[{\tilde{U}}_{0}\right]}_{\tilde{x}=0}=[({\tilde{U}}_{0}){,}_{\tilde{x}\tilde{x}}{]}_{\tilde{x}=0}=[({\tilde{U}}_{0}){,}_{\tilde{x}\tilde{x}}{]}_{\tilde{x}=1}=0,$$

(153) $$[\left(1-{\tilde{\gamma }\tilde{\omega }}^{2}\right)({\tilde{U}}_{0}){,}_{\tilde{x}}-{\tilde{l}}^{2}({\tilde{U}}_{0}){,}_{\tilde{x}\tilde{x}\tilde{x}}{]}_{\tilde{x}=1}={\tilde{F}}_{A}.$$

(154) $${\mathrm{Solution}\;\tilde{N}}_{0}:{\tilde{N}}_{0}\left(\tilde{x}\right)=\left(1-{\tilde{\gamma }\tilde{\omega }}^{2}\right)({\tilde{U}}_{0}){,}_{\tilde{x}}-{\tilde{l}}^{2}({\tilde{U}}_{0}){,}_{\tilde{x}\tilde{x}\tilde{x}}.$$

**Figure 2:**
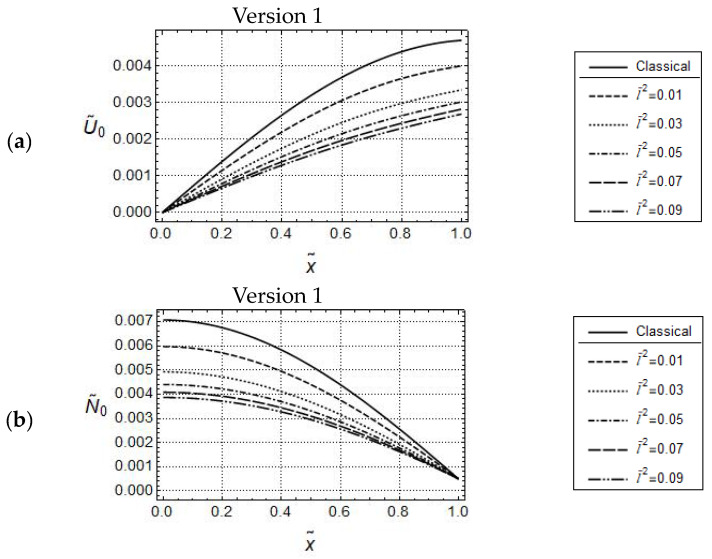
Force controlled loading. Model responses according to Version 1 for various values of $\tilde{l}$ with $\tilde{w}$ = 1.5, ${\tilde{F}}_{A}=5\cdot 10^{-4}\;;$ distributions of (**a**) ${\tilde{U}}_{0}$ and (**b**) ${\tilde{N}}_{0}$.

**Figure 3:**
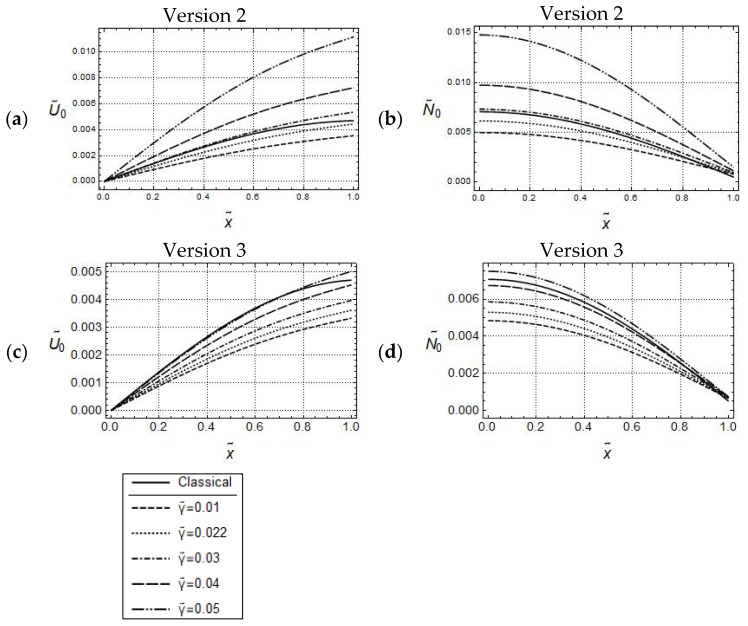
Force controlled loading. Model responses according to Version 2 (**top**) and Version 3 (**bottom**) for various values of $\tilde{\gamma }$ with $\tilde{w}$ = 1.5, ${\tilde{F}}_{A}=5\cdot 10^{-4}$ and ${\tilde{l}}^{2}=0.05$; (**a**,**c**) distributions of ${\tilde{U}}_{0}$ and (**b**,**d**) distributions of ${\tilde{N}}_{0}$.

Resulting distributions of ${\tilde{U}}_{0}$ and ${\tilde{N}}_{0}$ are illustrated in Figure 2 and Figure 3. In addition, corresponding distributions predicted by classical elasticity are shown in these figures.

It is well known that gradient elasticity includes parameters which control length-scale effects captured by the constitutive theory. In the case of Version 1 ($\tilde{\gamma }=0$), the only material parameter responsible for length scale effects is the internal material length $\tilde{l}$. Figure 2 illustrates the effect of $\tilde{l}$ on the predicted model responses for the case of Version 1. It can be seen that for small values of frequencies as, e.g., $\tilde{\omega }=1.5$, all distributions of ${\tilde{U}}_{0}$ are monotonically increasing, do not intersect for $\tilde{x}>0\;$ and indicate the gradient stiffening effect in comparison to the classical solution. The stiffening effect is increasing with increasing values of $\tilde{l}$. All corresponding ${\tilde{N}}_{0}$-distributions, shown in Figure 2b, are monotonically decreasing, do not intersect for $\tilde{x}<1$ and are below the classical one. Thus, the distributions of ${\tilde{U}}_{0}$ and ${\tilde{N}}_{0}$ indicate a common intersection point, respectively. 

No regular tendencies in the distributions of ${\tilde{U}}_{0}$ and ${\tilde{N}}_{0}$ can be recognized, or even the opposite may happen, for sufficiently large values of $\tilde{\omega }$. In particular, new intersection points in the graphs may occur and positions of intersection points can change depending on the applied frequency. Since the relationships for large values of $\tilde{\omega }$ are similar to those reported in Broese et al. [15], they will not be further discussed here.

In the case of Versions 2 and 3 ($\tilde{\gamma }\;\neq 0$), length scale effects can be controlled besides by $\tilde{l}$, also by $\tilde{\gamma }$ (see Figure 3). Apparently, for both versions, and for the sufficiently small frequency $\tilde{\omega }=1.5$, the ${\tilde{U}}_{0}$- and ${\tilde{N}}_{0}$-distributions, for $\tilde{l}$ = constant, look similar to the ones for Version 1 in Figure 2. Moreover, the ${\tilde{U}}_{0}$-distributions increase with increasing values of $\tilde{\gamma }$, and can exceed the one predicted by classical elasticity. This, in turn, indicates that, for $\tilde{l}$ = constant, the nonclassical acceleration terms controlled by $\tilde{\gamma }$, cause (dynamical) softening in the material behavior. However, it must be emphasized that the amounts of these distributions are considerably smaller for Version 3. This behavior carries over to the ${\tilde{N}}_{0}$-distributions as well. Altogether, the predicted responses by Version 2 and Version 3 are qualitatively similar to each other but, depending on the $\tilde{\gamma }$-values, significant quantitative differences can occur.

#### 5.1.3. Displacement Controlled Loading

All the boundary conditions and the solutions for ${\tilde{N}}_{0}$ are the same as in the last section, except for the boundary condition at $\tilde{x}=1$ for the sectional force $\tilde{N},$ which is now replaced by the displacement boundary condition $\tilde{U}\left(1,\tilde{t}\right)={\tilde{U}}_{A}e^{i\tilde{\omega }\tilde{t}},\;{\tilde{U}}_{A}$ = constant.

Versions 1, 3

(155) $$B.C.:{\left[{\tilde{U}}_{0}\right]}_{\tilde{x}=0}=[({\tilde{U}}_{0}){,}_{\tilde{x}\tilde{x}}{]}_{\tilde{x}=0}=[({\tilde{U}}_{0}){,}_{\tilde{x}\tilde{x}}{]}_{\tilde{x}=1}=0\;,\;{\left[{\tilde{U}}_{0}\right]}_{\tilde{x}=1}={\tilde{U}}_{A}$$

(156) $${\mathrm{Solution}\;\tilde{N}}_{0}:{\tilde{N}}_{0}\left(\tilde{x}\right)=({\tilde{U}}_{0}){,}_{\tilde{x}}-{\tilde{l}}^{2}({\tilde{U}}_{0}){,}_{\tilde{x}\tilde{x}\tilde{x}}.$$

Version 2

(157) $$B.C.:{\left[{\tilde{U}}_{0}\right]}_{\tilde{x}=0}=[({\tilde{U}}_{0}){,}_{\tilde{x}\tilde{x}}{]}_{\tilde{x}=0}=[({\tilde{U}}_{0}){,}_{\tilde{x}\tilde{x}}{]}_{\tilde{x}=1}=0\;,\;{\left[{\tilde{U}}_{0}\right]}_{\tilde{x}=1}={\tilde{U}}_{A}.$$

(158) $${\mathrm{Solution}\;\tilde{N}}_{0}:{\tilde{N}}_{0}\left(\tilde{x}\right)=\left(1-{\tilde{\gamma }\tilde{\omega }}^{2}\right)({\tilde{U}}_{0}){,}_{\tilde{x}}-{\tilde{l}}^{2}({\tilde{U}}_{0}){,}_{\tilde{x}\tilde{x}\tilde{x}}.$$

**Figure 4:**
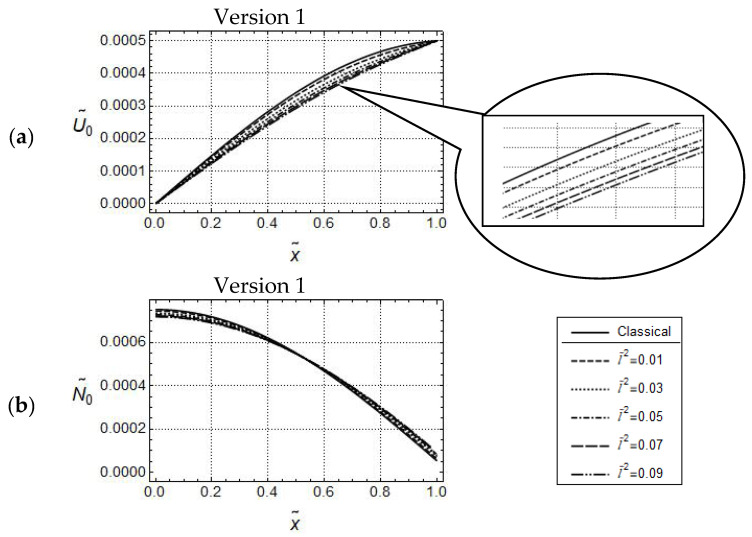
Displacement controlled loading. Model responses according to Version 1 for various values of $\tilde{l}$, with $\tilde{w}=1.5$, ${\tilde{U}}_{A}=5\cdot 10^{-3}$; (**a**) distributions of ${\tilde{U}}_{0}$ and (**b**) distributions of ${\tilde{N}}_{0}$.

**Figure 5:**
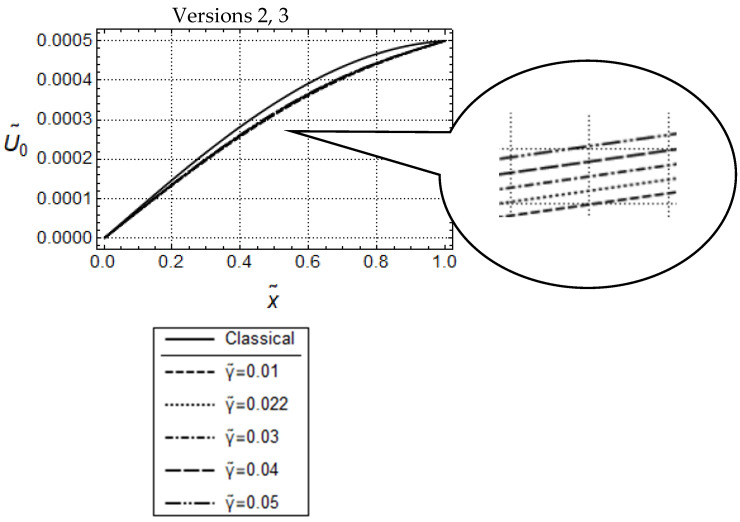
Displacement controlled loading. Identical ${\tilde{U}}_{0}$-distributions for Versions 2 and 3; $\tilde{w}$ = 1.5, ${\tilde{U}}_{A}=5\cdot 10^{-3}$, ${\tilde{l}}^{2}=0.04$.

**Figure 6:**
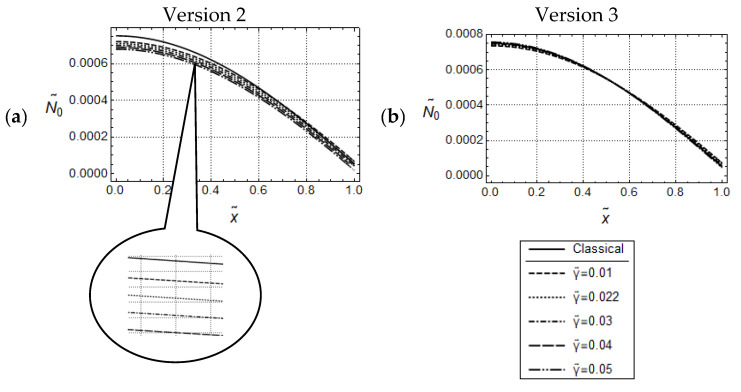
Displacement controlled loading. Predicted ${\tilde{N}}_{0}$-distributions for Version 2 (**a**) and Version 3 (**b**); $\tilde{w}$ = 1.5, ${\tilde{U}}_{A}=5\cdot 10^{-3}$, ${\tilde{l}}^{2}=0.04$.

Predicted distributions for Version 1 ($\tilde{\gamma }$ = 0) and classical elasticity for the above displacement-controlled boundary conditions are depicted in Figure 4 for frequency $\tilde{\omega }$ and ${\tilde{U}}_{A}=5\cdot 10^{-3}$. The general observations concerning ${\tilde{U}}_{0}$-responses are similar to those for force-controlled loading. Clearly, due to the imposed boundary conditions, the ${\tilde{U}}_{0}$-distributions intersect now at $\tilde{x}$ = 1 as well. It can be recognized from Figure 4a, that for the sufficiently small value $\tilde{\omega }=1.5$, only small quantitative differences are visible, which could be expected because of the assumed displacement boundary conditions. In the related ${\tilde{N}}_{0}$-distributions only small quantitative differences are visible as well (cf. Figure 4b). However, there is the remarkable qualitative difference that, now, these distributions do intersect for some $0<\tilde{x}<1.$ For sufficiently large values of $\tilde{\omega }$ no regular tendencies in the predicted responses can be stated. As the differential Equation (148) for ${\tilde{U}}_{0}$ and the associated boundary conditions (155) and (157) are identical for Versions 2 and 3, the predicted ${\tilde{U}}_{0}$-distributions according to both versions are identical as well. The graphs of ${\tilde{U}}_{0}$-distributions in Figure 5 attest, for the small frequency $\tilde{\omega }=1.5$, only small quantitative differences for various values of $\tilde{\gamma }$. After magnification of Figure 5, it becomes clear that these distributions increase with increasing values of $\tilde{\gamma }$ but are always below the one predicted by classical elasticity. On the other hand, observing closely the ${\tilde{N}}_{0}$-distributions in Figure 6, it becomes clear that these distributions for Version 2 decrease with increasing values of $\tilde{\gamma }$ and intersect the one predicted by classical elasticity for some $\tilde{x}\in \left(0,1\right)$. Comparison with ${\tilde{N}}_{0}$-distributions for Version 3, depicted in Figure 6b, reveal for Version 2 smaller values of ${\tilde{N}}_{0}$ than for Version 3. In addition, by increasing the value of $\tilde{\gamma }$, these distributions in Figure 6b are increasing. This observation is the opposite of the one made in Figure 6a.

Concluding this section, we can state that there are significant quantitative and qualitative differences for the considered tension/compression loading conditions.

### 5.2. Cantilever Beam under Dynamical Transverse Load

#### 5.2.1. Governing Equations

Consider, now, the cantilever beam to be loaded only transversely so that U≡0, and suppose that, ${\bar{q}}_{r}=q_{r}\equiv 0$. For this case, we find from Equation (113) (or from Equation (142)) that

(159) $$-\rho A\ddot{w}+\rho I{\ddot{w}}^{\prime \prime }-\gamma I\;{\ddot{w}}^{⁗}-{\mathrm{EIw}}^{⁗}+l^{2}{\mathrm{EI}\;w}^{\prime \prime \prime \prime \prime \prime }=0.$$

The dimensionless form of it reads

(160) $$-\tilde{A}\tilde{w}{,}_{\tilde{t}\tilde{t}}+\tilde{I}\tilde{w}{,}_{\tilde{t}\tilde{t}\tilde{x}\tilde{x}}-\tilde{\gamma }\tilde{I}\tilde{w}{,}_{\tilde{t}\tilde{t}\tilde{x}\tilde{x}\tilde{x}\tilde{x}}-\tilde{I}\tilde{w}{,}_{\tilde{x}\tilde{x}\tilde{x}\tilde{x}}+{\tilde{l}}^{2}\tilde{I}\tilde{w}{,}_{\tilde{x}\tilde{x}\tilde{x}\tilde{x}\tilde{x}\tilde{x}}=0.$$

Obviously, for $\tilde{\gamma }=0$, Equation (160) represents the equation of motion for Version 1, and for $\tilde{\gamma }\neq 0$ it represents the equation of motion for Versions 2, 3. We assume that, at $x_{1}=L$, the beam is subject to transverse load, force or deflection controlled, of the form ${\mathrm{Be}}^{\omega \mathrm{it}}$, where again ω is an operating frequency, B is a force-or deflection-like amplitude and i is the imaginary unit. This suggests for the solution of Equation (160) to make the Ansatz

(161) $$\tilde{w}\left(\tilde{x},\;\tilde{t}\right)={\tilde{w}}_{0}\left(\tilde{x}\right)e^{i\tilde{\omega }\tilde{t}}.$$

After substitution of (161) into (160), and elimination of the factor $e^{i\tilde{\omega }\tilde{t}}$, we arrive at

(162) $${\tilde{A}\tilde{\omega }}^{2}{\tilde{w}}_{0}-{\tilde{\omega }}^{2}\tilde{Ι}({\tilde{w}}_{0}){,}_{\tilde{x}\tilde{x}}+\tilde{Ι}\left({\tilde{\gamma }\tilde{\omega }}^{2}-1\right)({\tilde{w}}_{0}){,}_{\tilde{x}\tilde{x}\tilde{x}\tilde{x}}+{\tilde{l}}^{2}\tilde{Ι}({\tilde{w}}_{0}){,}_{\tilde{x}\tilde{x}\tilde{x}\tilde{x}\tilde{x}\tilde{x}}=0,$$

which, along with a set of boundary conditions, can be solved by employing standard methods. Distribution of sectional forces can be established by inserting into corresponding formulae the solutions $\tilde{w}\left(\tilde{x},\;\tilde{t}\right)$. In the subsequent sections, we will discuss force and deflection controlled bending of the cantilever beam.

#### 5.2.2. Force Controlled Bending

The manipulations in the current and the next section are similar to those in Section 5.1.2 and Section 5.1.3, respectively. We suppose for Versions 1, 3 the boundary conditions $w\left(0,t\right)=0$, $w^{\prime }\left(0,\;t\right)=0$, $m_{r}\left(0,\;t\right)=m_{r}\left(L,\;t\right)=0$, $M_{r}\left(L,\;t\right)=0$ and $V\left(L,t\right)=V_{r}\left(L,t\right)=F_{A}e^{i\omega t}$, where again $F_{A}=\mathrm{constant}$ is a force amplitude. From these boundary conditions, we can deduce boundary conditions for the differential Equation (162) by taking into account Equations (135)–(138) and (161), and eliminating the factor $e^{i\tilde{\omega }\tilde{t}}$. In particular, the boundary condition for $\tilde{V}$ becomes $\tilde{V}\left(1,\;\tilde{t}\right)={\tilde{F}}_{A}e^{i\tilde{\omega }\tilde{t}}$, where now ${\tilde{F}}_{A}:=\frac{F_{A}}{EL^{2}}$. Also, we can deduce from Equations (135) and (137) that, for Versions 1, 3, $\tilde{V}={\tilde{V}}_{0}\left(\tilde{x}\right)e^{i\tilde{\omega }\tilde{t}}$, $\tilde{M}={\tilde{M}}_{0}\left(\tilde{x}\right)e^{i\tilde{\omega }\tilde{t}}$, with ${\tilde{V}}_{0}\left(\tilde{x}\right)=-{\tilde{\omega }}^{2}\tilde{Ι}\left({\tilde{w}}_{0}\right){,}_{\tilde{x}}$ + $\tilde{I}\left({\tilde{\gamma }\tilde{\omega }}^{2}-1\right)\left({\tilde{w}}_{0}\right){,}_{\tilde{x}\tilde{x}\tilde{x}}+{\tilde{l}}^{2}\tilde{I}\left({\tilde{w}}_{0}\right){,}_{\tilde{x}\tilde{x}\tilde{x}\tilde{x}\tilde{x}}$ and ${\tilde{M}}_{0}\left(\tilde{x}\right)=-\tilde{Ι}\left({\tilde{w}}_{0}\right){,}_{\tilde{x}\tilde{x}}+{\tilde{l}}^{2}\;\tilde{I}\left({\tilde{w}}_{0}\right){,}_{\tilde{x}\tilde{x}\tilde{x}\tilde{x}}$.

With the help of Equation (106)–(108), and using similar manipulations as for Versions 1, 3, we can deduce corresponding boundary conditions and distributions ${\tilde{V}}_{0}$, ${\tilde{M}}_{0}$ for Version 2. Altogether, we have the following.

Versions 1, 3

(163) $$B.C.:{\left[{\tilde{w}}_{0}\right]}_{\tilde{x}=0}=\left[({\tilde{w}}_{0}\right){,}_{\tilde{x}}{]}_{\tilde{x}=0}=\left[({\tilde{w}}_{0}\right){,}_{\tilde{x}\tilde{x}\tilde{x}}{]}_{\tilde{x}=0}=\left[({\tilde{w}}_{0}\right){,}_{\tilde{x}\tilde{x}\tilde{x}}{]}_{\tilde{x}=1}\;=\left[({\tilde{w}}_{0}\right){,}_{\tilde{x}\tilde{x}}-{\tilde{l}}^{2}({\tilde{w}}_{0}){,}_{\tilde{x}\tilde{x}\tilde{x}\tilde{x}}{]}_{\tilde{x}=1}=0,$$

(164) $${\left[-{\tilde{\omega }}^{2}\tilde{Ι}\left({\tilde{w}}_{0}\right){,}_{\tilde{x}}+\tilde{Ι}\left({\tilde{\gamma }\tilde{\omega }}^{2}-1\right)\left({\tilde{w}}_{0}\right){,}_{\tilde{x}\tilde{x}\tilde{x}}+{\tilde{l}}^{2}\tilde{I}\left({\tilde{w}}_{0}\right){,}_{\tilde{x}\tilde{x}\tilde{x}\tilde{x}\tilde{x}}\right]}_{\tilde{x}=1}={\tilde{F}}_{A}.$$

(165) $${\mathrm{Solution}\;\tilde{V}}_{0}:{\tilde{V}}_{0}\left(\tilde{x}\right)=-{\tilde{\omega }}^{2}\tilde{Ι}\left({\tilde{w}}_{0}\right){,}_{\tilde{x}}+\tilde{Ι}\left({\tilde{\gamma }\tilde{\omega }}^{2}-1\right)\left({\tilde{w}}_{0}\right){,}_{\tilde{x}\tilde{x}\tilde{x}}+{\tilde{l}}^{2}\tilde{I}\left({\tilde{w}}_{0}\right){,}_{\tilde{x}\tilde{x}\tilde{x}\tilde{x}\tilde{x}}.$$

(166) $${\mathrm{Solution}\;\tilde{M}}_{0}:{\tilde{M}}_{0}\left(\tilde{x}\right)=-\tilde{Ι}\left({\tilde{w}}_{0}\right){,}_{\tilde{x}\tilde{x}}+{\tilde{l}}^{2}\tilde{I}\left({\tilde{w}}_{0}\right){,}_{\tilde{x}\tilde{x}\tilde{x}\tilde{x}}.$$

Version 2

(167) $$B.C.:{\left[{\tilde{w}}_{0}\right]}_{\tilde{x}=0}=\left[({\tilde{w}}_{0}\right){,}_{\tilde{x}}{]}_{\tilde{x}=0}=\left[({\tilde{w}}_{0}\right){,}_{\tilde{x}\tilde{x}\tilde{x}}{]}_{\tilde{x}=0}=\left[({\tilde{w}}_{0}\right){,}_{\tilde{x}\tilde{x}\tilde{x}}{]}_{\tilde{x}=1}\;=[\tilde{Ι}\left({\tilde{\gamma }\tilde{\omega }}^{2}-1\right)({\tilde{w}}_{0}){,}_{\tilde{x}\tilde{x}}+{\tilde{l}}^{2}({\tilde{w}}_{0}){,}_{\tilde{x}\tilde{x}\tilde{x}}{]}_{\tilde{x}=1}=0,$$

(168) $${\left[-{\tilde{\omega }}^{2}\tilde{Ι}\left({\tilde{w}}_{0}\right){,}_{\tilde{x}}+\tilde{Ι}\left({\tilde{\gamma }\tilde{\omega }}^{2}-1\right)\left({\tilde{w}}_{0}\right){,}_{\tilde{x}\tilde{x}\tilde{x}}+{\tilde{l}}^{2}\tilde{I}\left({\tilde{w}}_{0}\right){,}_{\tilde{x}\tilde{x}\tilde{x}\tilde{x}\tilde{x}}\right]}_{\tilde{x}=1}={\tilde{F}}_{A}.$$

(169) $${\mathrm{Solution}\;\tilde{V}}_{0}:{\tilde{V}}_{0}\left(\tilde{x}\right)=-{\tilde{\omega }}^{2}\tilde{Ι}\left({\tilde{w}}_{0}\right){,}_{\tilde{x}}+\tilde{Ι}\left({\tilde{\gamma }\tilde{\omega }}^{2}-1\right)\left({\tilde{w}}_{0}\right){,}_{\tilde{x}\tilde{x}\tilde{x}}+{\tilde{l}}^{2}\tilde{I}\left({\tilde{w}}_{0}\right){,}_{\tilde{x}\tilde{x}\tilde{x}\tilde{x}\tilde{x}}.$$

(170) $${\mathrm{Solution}\;\tilde{M}}_{0}:{\tilde{M}}_{0}\left(\tilde{x}\right)=-\tilde{Ι}\left(1-{\tilde{\gamma }\tilde{\omega }}^{2}\right)\left({\tilde{w}}_{0}\right){,}_{\tilde{x}\tilde{x}}+{\tilde{l}}^{2}\tilde{I}\left({\tilde{w}}_{0}\right){,}_{\tilde{x}\tilde{x}\tilde{x}\tilde{x}}.$$

**Figure 7:**
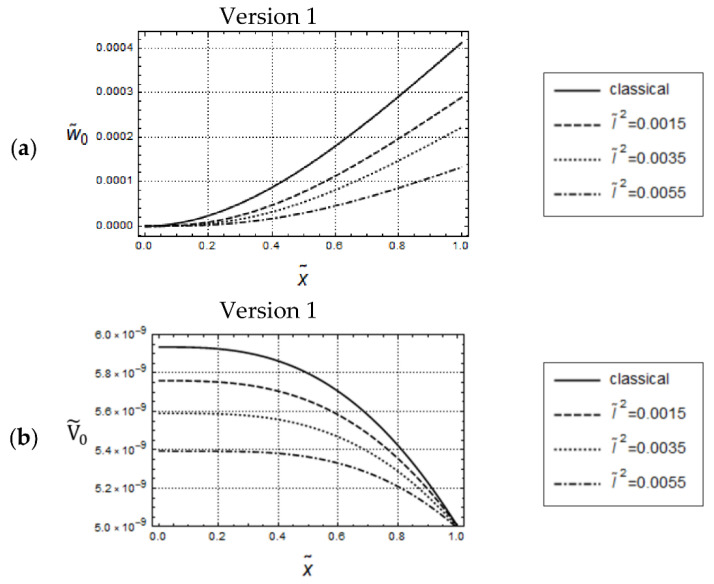
Force control loading. Model responses according to Version 1 for various values of $\tilde{l}$ and $\tilde{w}$ = 0.05, ${\tilde{F}}_{A}=5\cdot 10^{-9}$. Distributions of (**a**) ${\tilde{w}}_{0}$ and (**b**) ${\tilde{V}}_{0}$.

By comparing Figure 7 with Figure 2, it can be recognized that the ${\tilde{w}}_{0}$- and ${\tilde{V}}_{0}$-distributions are essentially similar to the ${\tilde{U}}_{0}$- and ${\tilde{N}}_{0}$-distributions, respectively, the only difference being that the ${\tilde{w}}_{0}$-distributions are convex whereas the ${\tilde{U}}_{0}$-distributions are concave.

**Figure 8:**
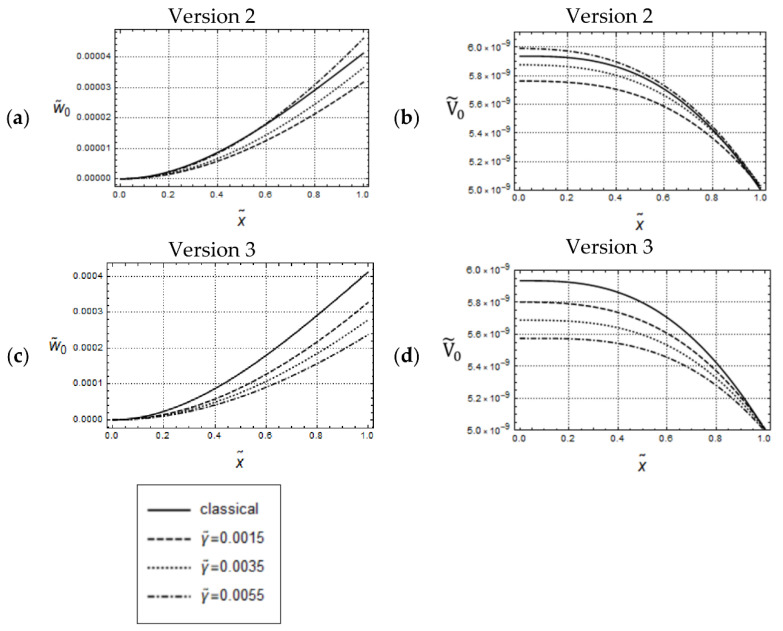
Force controlled loading. Model responses according to Version 2 (**top**) and Version 3 (**bottom**) for various values of $\tilde{\gamma }$ and $\tilde{w}$ = 0.05, ${\tilde{F}}_{A}=5\cdot 10^{-9}$, $\tilde{l}=0.05$; (**a**,**c**) distributions of ${\tilde{w}}_{0}$ and (**b**,**d**) distributions of ${\tilde{V}}_{0}$.

Also, similar to the uniaxial loading cases in Figure 3a,c, for the small frequency $\tilde{\omega }=0.05$, and for keeping $\tilde{l}$ constant, by increasing values of $\tilde{\gamma }$, the ${\tilde{w}}_{0}$-distributions predicted by Versions 2, 3 are increasing, which indicates that dynamic gradient softening can occur (see Figure 8a,c). However, the amounts of the ${\tilde{w}}_{0}$-distributions predicted by Version 3 are smaller than those predicted by Version 2. Furthermore, for forced controlled loadings, in the case of Version 3, there is a noticeable qualitative difference between the ${\tilde{U}}_{0}$-distributions in Figure 3c and the ${\tilde{w}}_{0}$-distributions in Figure 8c: All ${\tilde{w}}_{0}$-distributions are bounded from above by the classical one, in opposite to the ${\tilde{U}}_{0}$-distributions.

#### 5.2.3. Deflection Controlled Bending

All the boundary conditions and the solutions ${\tilde{V}}_{0}$, ${\tilde{M}}_{0}$ are the same as in the last section, except for the boundary conditions at $\tilde{x}=1$ for the sectional forces $\tilde{V}$, which are now replaced by the deflection boundary conditions $\tilde{w}\left(1,\;\tilde{t}\right)={\tilde{w}}_{A}e^{i\tilde{\omega }\tilde{t}}$, ${\tilde{w}}_{A}=\mathrm{constant}$.

Versions 1, 3

(171) $$B.C.:{\left[{\tilde{w}}_{0}\right]}_{\tilde{x}=0}=\left[({\tilde{w}}_{0}\right){,}_{\tilde{x}}{]}_{\tilde{x}=0}=\left[({\tilde{w}}_{0}\right){,}_{\tilde{x}\tilde{x}\tilde{x}}{]}_{\tilde{x}=0}=\left[({\tilde{w}}_{0}\right){,}_{\tilde{x}\tilde{x}\tilde{x}}{]}_{\tilde{x}=1}\;=\left[({\tilde{w}}_{0}\right){,}_{\tilde{x}\tilde{x}}-{\tilde{l}}^{2}({\tilde{w}}_{0}){,}_{\tilde{x}\tilde{x}\tilde{x}\tilde{x}}{]}_{\tilde{x}=1}=0,\;{\left[{\tilde{w}}_{0}\right]}_{\tilde{x}=1}={\tilde{w}}_{A}.$$

(172) $${\mathrm{Solution}\;\tilde{V}}_{0}:{\tilde{V}}_{0}\left(\tilde{x}\right)=-{\tilde{\omega }}^{2}\tilde{Ι}\left({\tilde{w}}_{0}\right){,}_{\tilde{x}}+\tilde{Ι}\left({\tilde{\gamma }\tilde{\omega }}^{2}-1\right)\left({\tilde{w}}_{0}\right){,}_{\tilde{x}\tilde{x}\tilde{x}}+{\tilde{l}}^{2}\tilde{I}\left({\tilde{w}}_{0}\right){,}_{\tilde{x}\tilde{x}\tilde{x}\tilde{x}\tilde{x}}.$$

(173) $${\mathrm{Solution}\;\tilde{M}}_{0}:{\tilde{M}}_{0}\left(\tilde{x}\right)=-\tilde{Ι}\left({\tilde{w}}_{0}\right){,}_{\tilde{x}\tilde{x}}+{\tilde{l}}^{2}\tilde{I}\left({\tilde{w}}_{0}\right){,}_{\tilde{x}\tilde{x}\tilde{x}\tilde{x}}.$$

Version 2

(174) $$B.C.:{\left[{\tilde{w}}_{0}\right]}_{\tilde{x}=0}=\left[({\tilde{w}}_{0}\right){,}_{\tilde{x}}{]}_{\tilde{x}=0}=\left[({\tilde{w}}_{0}\right){,}_{\tilde{x}\tilde{x}\tilde{x}}{]}_{\tilde{x}=0}=[({\tilde{w}}_{0}){,}_{\tilde{x}\tilde{x}\tilde{x}}{]}_{\tilde{x}=1}\;=[(\tilde{Ι}\left({\tilde{\gamma }\tilde{\omega }}^{2}-1\right)({\tilde{w}}_{0}){,}_{\tilde{x}\tilde{x}}+{\tilde{l}}^{2}({\tilde{w}}_{0}){,}_{\tilde{x}\tilde{x}\tilde{x}\tilde{x}}{]}_{\tilde{x}=1},\;{\left[{\tilde{w}}_{0}\right]}_{\tilde{x}=1}={\tilde{w}}_{A}.$$

(175) $${\mathrm{Solution}\;\tilde{V}}_{0}:{\tilde{V}}_{0}\left(\tilde{x}\right)=-{\tilde{\omega }}^{2}\tilde{Ι}\left({\tilde{w}}_{0}\right){,}_{\tilde{x}}+\tilde{Ι}\left({\tilde{\gamma }\tilde{\omega }}^{2}-1\right)\left({\tilde{w}}_{0}\right){,}_{\tilde{x}\tilde{x}\tilde{x}}+{\tilde{l}}^{2}\tilde{I}\left({\tilde{w}}_{0}\right){,}_{\tilde{x}\tilde{x}\tilde{x}\tilde{x}\tilde{x}}.$$

(176) $${\mathrm{Solution}\;\tilde{M}}_{0}:{\tilde{M}}_{0}\left(\tilde{x}\right)=-\tilde{Ι}\left(1-{\tilde{\gamma }\tilde{\omega }}^{2}\right)+{\tilde{l}}^{2}\tilde{I}\left({\tilde{w}}_{0}\right){,}_{\tilde{x}\tilde{x}\tilde{x}\tilde{x}}.$$

**Figure 9:**
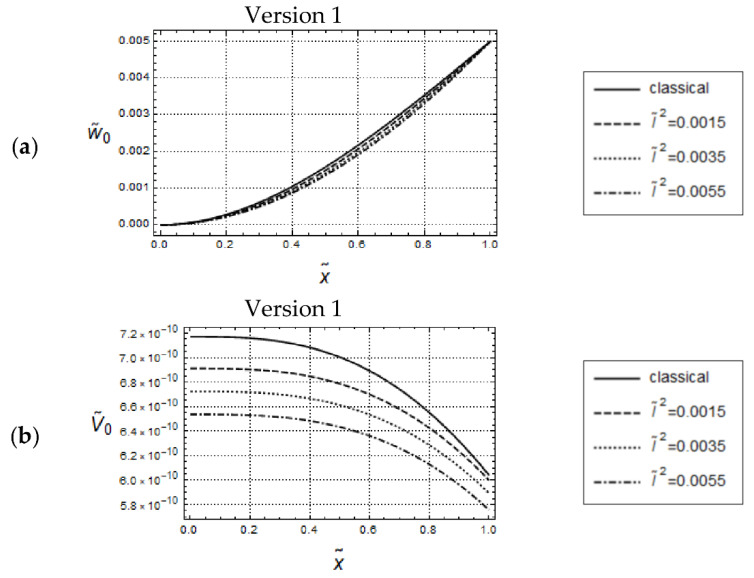
Deflection controlled loading. Model responses according to Version 1 for various values of $\tilde{l}$ and $\tilde{w}$ = 0.05, ${\tilde{w}}_{A}=5\cdot 10^{-3}$; distributions of (**a**) ${\tilde{w}}_{0}$ and (**b**) ${\tilde{V}}_{0}$.

**Figure 10:**
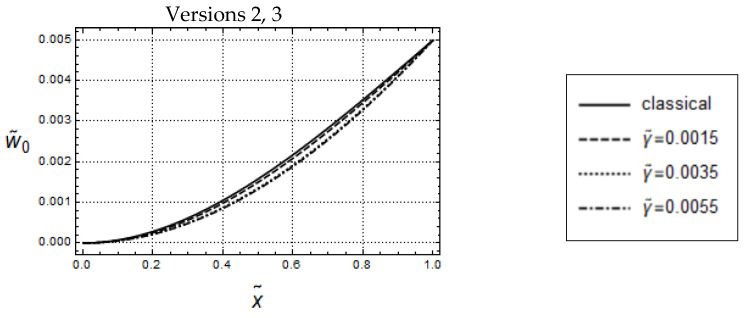
Displacement controlled loading. Identical ${\tilde{w}}_{0}$-distributions for Versions 2 and 3; $\tilde{w}$ = 0.05, ${\tilde{U}}_{A}=5\cdot 10^{-3}$, ${\tilde{l}}^{2}=0.04$.

**Figure 11:**
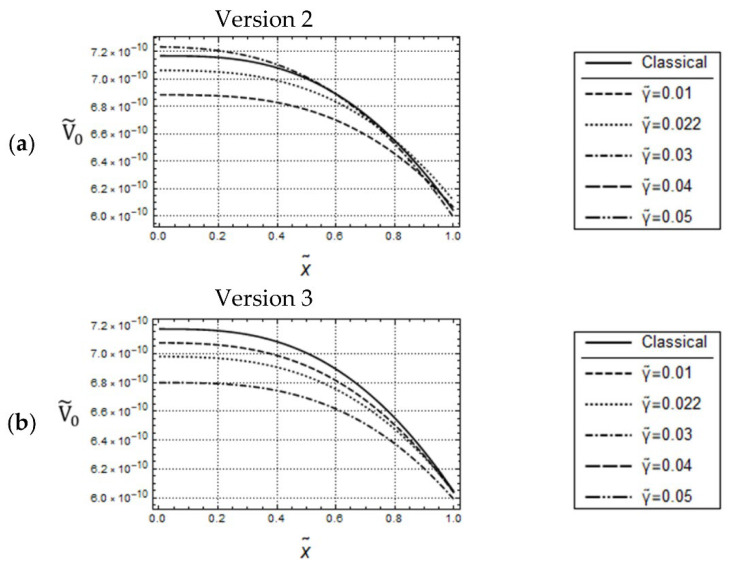
Deflection controlled loading. Predicted ${\tilde{V}}_{0}$-distributions for Version 2 (**a**) and Version 3 (**b**); $\tilde{w}$ = 0.05, ${\tilde{U}}_{A}=5\cdot 10^{-3}$, ${\tilde{l}}^{2}=0.04$.

Figure 9 illustrates predicted ${\tilde{w}}_{0}$- and ${\tilde{V}}_{0}$-distributions according to Version 1 ($\tilde{\gamma }=0)$ and classical elasticity for $\tilde{w}$ = 0.05 and ${\tilde{w}}_{A}=5\cdot 10^{-3}$. Except for the differences in the curvatures, the ${\tilde{w}}_{0}$– distributions in Figure 9a look similar to the ${\tilde{U}}_{0}$-distributions in Figure 4a. However, there are significant qualitative and quantitative differences between the ${\tilde{V}}_{0}$-distributions in Figure 9b and the ${\tilde{N}}_{0}$-distributions in Figure 4b. The most important one is that there are no intersections between the graphs of the curves in Figure 9b. The remarks concerning the predicted responses according to Versions 2, 3 in Figure 10 and Figure 11 are similar to those for the uniaxial loadings in Figure 5 and Figure 6.

The main conclusion which can be drawn from the calculated responses in the present and the last section is that, depending on the imposed boundary conditions, both similarities and differences in the predicted distributions due to uniaxial and bending loadings may occur. The most important observation, however, is that if γ ≠ 0, then significant qualitative and quantitative differences in the predicted responses can occur, depending on whether acceleration terms are present in the boundary conditions or not.

## 6. Concluding Remarks

The explicit gradient elasticity proposed by Mindlin [7] was an important step toward modeling nonlocalities and length scale effects in the material response. The original formulation of this theory is characterized by the presence of acceleration terms in the boundary tractions. It was argued in [15] that such boundary tractions are physically unacceptable as they are not objective. The argumentation against boundary tractions including acceleration terms has been extended in the present paper within the context of the principle of material frame indifference. It is shown that if this principle is assumed to hold, then boundary tractions including acceleration terms are not acceptable since either these tractions will not be objective, or the response functions of the associated stresses will not satisfy the principle of material frame indifference. The differences in the model responses, according to the two different forms of boundary tractions, are not negligible. In fact, it has been demonstrated with reference to uniaxial and bending loadings, that significant qualitative and quantitative differences may occur between the corresponding model responses. Different to [15], homogenous nonclassical boundary tractions are assumed here in the calculated examples, which, for the time being, seems to be physically more realistic. Besides force controlled loading histories, displacement controlled ones have also been considered and compared with each other. Thus, the present paper, compared with the analysis given [9,10], offers a more comprehensive discussion of the boundary conditions, and in addition, for the first time, it provides a consistent Euler–Bernoulli beam theory for bending in dynamics.

## Data Availability

The original contributions presented in the study are included in the article, further inquiries can be directed to the corresponding authors.

## Appendix A

Substitution of Equation (56) into Equations (89) and (91) gives

(A1) $$\overset{\;}{\underset{V}{\int }}\left(\partial_{1}\Sigma_{11}^{\left(t\right)}\right)\delta \mathrm{UdV}+\overset{\;}{\underset{V}{\int }}\left(\partial_{1}\Sigma_{11}^{\left(t\right)}\right)x_{3}\delta \left(-\;w^{\prime }\right)\mathrm{dV}+\overset{\;}{\underset{V}{\int }}\left(\partial_{3}\Sigma_{31}^{\left(t\right)}\right)\delta \mathrm{UdV}\;\overset{\;}{\underset{V}{\int }}\left(\partial_{1}\Sigma_{11}^{\left(t\right)}\right)\delta \mathrm{UdV}+\overset{\;}{\underset{V}{\int }}\left(\partial_{1}\Sigma_{11}^{\left(t\right)}\right)x_{3}\delta \left(-w^{\prime }\right)\mathrm{dV}+\overset{\;}{\underset{V}{\int }}\left(\partial_{3}\Sigma_{31}^{\left(t\right)}\right)\delta \mathrm{UdV}\;-\overset{\;}{\underset{V}{\int }}\rho \left({\ddot{u}}_{1}{\delta u}_{1}+{\ddot{u}}_{3}{\delta u}_{3}\right)\mathrm{dV}=0.$$

The first term can be calculated with the aid of the constitutive law (58), (59) and (89) and the definitions (92):

(A2) $$\overset{\;}{\underset{V}{\int }}\left(\partial_{1}\Sigma_{11}^{\left(t\right)}\right)\delta \mathrm{UdV}=\overset{\;}{\underset{V}{\int }}\partial_{1}\left(\Sigma_{11}^{\left(t\right)}\delta U\right)\mathrm{dV}-\overset{\;}{\underset{V}{\int }}\Sigma_{11}^{\left(t\right)}\delta U^{\prime }\mathrm{dV}\;=\overset{L}{\underset{0}{\int }}\partial_{1}\left({\bar{N}}_{r}\delta U\right){\mathrm{dx}}_{1}-\overset{\;}{\underset{V}{\int }}\Sigma_{11}\delta U^{\prime }\mathrm{dV}-\overset{\;}{\underset{V}{\int }}\gamma \left({\ddot{U}}^{\prime }-{\ddot{w}}^{\prime \prime }x_{3}\right)\delta U^{\prime }\mathrm{dV}\;={\left[{\bar{N}}_{r}\;\delta U\right]}_{x_{1}=0}^{x_{1}=L}-\overset{\;}{\underset{V}{\int }}{E\varepsilon }_{11}\delta U^{\prime }\mathrm{dV}+\overset{\;}{\underset{V}{\int }}{\mu^{\prime }}_{111}\delta U^{\prime }\mathrm{dV}-\overset{L}{\underset{0}{\int }}\gamma A{\ddot{U}}^{\prime }{\delta U^{\prime }\mathrm{dx}}_{1}\;={\left[{\bar{N}}_{r}\;\delta U\right]}_{x_{1}=0}^{x_{1}=L}-\overset{\;}{\underset{V}{\int }}{E\varepsilon }_{11}\delta U^{\prime }\mathrm{dV}+\overset{\;}{\underset{V}{\int }}\partial_{1}\overset{\;}{\underset{A}{\int }}\mu_{111}{\mathrm{dS}\;\delta U^{\prime }\mathrm{dx}}_{1}-\overset{\;}{\underset{V}{\int }}\mu_{111}\delta U^{\prime \prime }\mathrm{dV}\;-\overset{L}{\underset{0}{\int }}\gamma A{\ddot{U}}^{\prime }{\delta U^{\prime }\mathrm{dx}}_{1}\;={\left[{\bar{N}}_{r}\;\delta U\right]}_{x_{1}=0}^{x_{1}=L}+{\left[{\bar{H}}_{r}\;\delta U^{\prime }\right]}_{x_{1}=0}^{x_{1}=L}-\overset{\;}{\underset{V}{\int }}\left({E\varepsilon }_{11}\delta U^{\prime }+\mu_{111}\delta U^{\prime \prime }\right)\mathrm{dV}-\overset{L}{\underset{0}{\int }}\gamma A{\ddot{U}}^{\prime }{\delta U^{\prime }\mathrm{dx}}_{1}.$$

The second term in Equation (A1) can be evaluated in a similar fashion:

(A3) $$\overset{\;}{\underset{V}{\int }}\left(\partial_{1}\Sigma_{11}^{\left(t\right)}x_{3}\right)\delta \left(-w^{\prime }\right)\mathrm{dV}=\overset{\;}{\underset{V}{\int }}\partial_{1}\left[\Sigma_{11}^{\left(t\right)}x_{3}\delta \left(-w^{\prime }\right)\right]\mathrm{dV}-\overset{\;}{\underset{V}{\int }}\Sigma_{11}^{\left(t\right)}x_{3}\delta \left(-w^{\prime \prime }\right)\mathrm{dV}\;=\overset{L}{\underset{0}{\int }}\partial_{1}\left[{\bar{M}}_{r}\delta \left(-w^{\prime }\right)\right]{\mathrm{dx}}_{1}-\overset{\;}{\underset{V}{\int }}{E\varepsilon }_{11}x_{3}\delta \left(-w^{\prime \prime }\right)\mathrm{dV}+\overset{\;}{\underset{V}{\int }}{\mu^{\prime }}_{111}x_{3}\delta \left(-w^{\prime \prime }\right)\mathrm{dV}\;-\overset{\;}{\underset{V}{\int }}\gamma \left(\ddot{U}-{\ddot{w}}^{\prime \prime }x_{3}\right)x_{3}\delta \left(-w^{\prime \prime }\right)\mathrm{dV}\;={\left[{\bar{M}}_{r}\;\delta \left(-w^{\prime }\right)\right]}_{x_{1}=0}^{x_{1}=L}+{\left[{\bar{m}}_{r}\;\delta \left(-w^{\prime \prime }\right)\right]}_{x_{1}=0}^{x_{1}=L}-\overset{\;}{\underset{V}{\int }}\left[{E\varepsilon }_{11}x_{3}\delta \left(-w^{\prime \prime }\right)+\mu_{111}\delta \left(-w^{\prime \prime \prime }\right)\right]\mathrm{dV}\;-\overset{L}{\underset{0}{\int }}\gamma I{\ddot{w}}^{\prime \prime }{\delta w^{\prime \prime }\mathrm{dx}}_{1}.\;$$

The third term in Equation (A1) gives

(A4) $$\overset{\;}{\underset{V}{\int }}\left(\partial_{3}\Sigma_{31}^{\left(t\right)}\right)\delta \mathrm{UdV}=\overset{L}{\underset{0}{\int }}2b{\left[\Sigma_{31}^{\left(t\right)}\right]}_{x_{3}=-c}^{x_{3}=c}{\delta \mathrm{Udx}}_{1}=\overset{L}{\underset{0}{\int }}{\bar{p}}_{r}{\delta \mathrm{Udx}}_{1}.$$

For the other integrals in Equation (A1) including stress components, we have

(A5) $$\overset{\;}{\underset{V}{\int }}\left(\partial_{3}\Sigma_{33}^{\left(t\right)}\right)\delta \mathrm{wdV}+\overset{\;}{\underset{V}{\int }}\left(\partial_{3}\Sigma_{13}^{\left(t\right)}\right)x_{3}\delta \left(-w^{\prime }\right)\mathrm{dV}+\overset{\;}{\underset{V}{\int }}\left(\partial_{1}\Sigma_{13}^{\left(t\right)}\right)\delta \mathrm{wdV}\;\overset{L}{\underset{0}{\int }}2b{\left[\Sigma_{33}\right]}_{x_{3}=-c}^{x_{3}=c}{\;\delta \mathrm{wdx}}_{1}-\overset{L}{\underset{0}{\int }}\partial_{1}\;\left\{\overset{\;}{\underset{A}{\int }}\left(\partial_{3}\Sigma_{31}^{\left(t\right)}\right)x_{3}\mathrm{dS}\;\delta w\right\}{\mathrm{dx}}_{1}\;+\overset{L}{\underset{0}{\int }}\left(\partial_{1}\overset{\;}{\underset{A}{\int }}\left(\partial_{3}\Sigma_{31}^{\left(t\right)}\right)x_{3}\mathrm{dS}\right){\delta \mathrm{wdx}}_{1}+\overset{L}{\underset{0}{\int }}\overset{\;}{\underset{A}{\int }}\left(\partial_{1}\Sigma_{13}^{\left(t\right)}\right){\delta w\;\mathrm{dS}\delta x}_{1}\;=\overset{L}{\underset{0}{\int }}2b{\left[\Sigma_{33}\right]}_{x_{3}=-c}^{x_{3}=c}{\;\delta \mathrm{wdx}}_{1}+{\left[{\bar{V}}_{r}{\;\delta w\;\mathrm{dx}}_{1}\right]}_{x_{1}=0}^{x_{1}=L}\;+\overset{L}{\underset{0}{\int }}\left(\partial_{1}\overset{\;}{\underset{A}{\int }}\left[\partial_{3}\left(\Sigma_{13}-\gamma {\ddot{w}}^{\prime }\right)x_{3}\mathrm{dS}\right]\right){\delta \mathrm{wdx}}_{1}+\overset{L}{\underset{0}{\int }}\left(\partial_{1}\overset{\;}{\underset{A}{\int }}\left(\Sigma_{13}+\gamma {\ddot{w}}^{\prime }\right)\mathrm{dS}\right){\delta \mathrm{wdx}}_{1}\;=\overset{L}{\underset{0}{\int }}2b{\left[\Sigma_{33}\right]}_{x_{3}=-c}^{x_{3}=c}{\;\delta \mathrm{wdx}}_{1}+{\left[{\bar{V}}_{r}{\;\delta w\;\mathrm{dx}}_{1}\right]}_{x_{1}=0}^{x_{1}=L}\;+\overset{L}{\underset{0}{\int }}\left(\partial_{1}\overset{\;}{\underset{A}{\int }}\left(\partial_{3}{\;\Sigma }_{13}\right)x_{3}\mathrm{dS}\right){\delta \mathrm{wdx}}_{1}+\overset{L}{\underset{0}{\int }}\left(\partial_{1}\overset{\;}{\underset{A}{\int }}\Sigma_{13}\mathrm{dS}\right){\delta \mathrm{wdx}}_{1}+\overset{L}{\underset{0}{\int }}\gamma A\;{\ddot{w}}^{\prime \prime }{\delta \mathrm{wdx}}_{1}\;={\left[{\bar{V}}_{r}\delta w\right]}_{x_{1}=0}^{x_{1}=L}+\overset{L}{\underset{0}{\int }}\left(2b{\left[\Sigma_{33}\right]}_{x_{3}=-c}^{x_{3}=c}+2b{\left[\partial_{1}\Sigma_{13}\right]}_{x_{3}=-c}^{x_{3}=c}+\gamma A\;{\ddot{w}}^{\prime \prime }\right){\delta \mathrm{wdx}}_{1}\;={\left[{\bar{V}}_{r}\delta w\right]}_{x_{1}=0}^{x_{1}=L}+\overset{L}{\underset{0}{\int }}{\bar{q}}_{r}{\delta \mathrm{wdx}}_{1}.$$

Keeping in mind the relations (56) for the displacement components, the last integral in Equation (A1), including acceleration terms, becomes

(A6) $$-\overset{\;}{\underset{V}{\int }}\rho \left({\ddot{u}}_{1}{\delta u}_{1}+{\ddot{u}}_{3}{\delta u}_{3}\right)\mathrm{dV}=-\overset{L}{\underset{0}{\int }}\left(\rho A\ddot{U}\delta U+\rho I{\ddot{w}}^{\prime }\delta w^{\prime }+\rho A\ddot{w}\delta w\right){\mathrm{dx}}_{1},\;$$

where, once more, partial integration has been applied.

Summing the results (A2)–(A6), and collecting terms, we may rewrite Equation (A1) in the following form:

(A7) $${\left[{\bar{N}}_{r}\delta U\right]}_{x_{1}=0}^{x_{1}=L}+{\left[{\bar{H}}_{r}\delta U^{\prime }\right]}_{x_{1}=0}^{x_{1}=L}+{\left[{\bar{V}}_{r}\delta w\right]}_{x_{1}=0}^{x_{1}=L}+{\left[{\bar{M}}_{r}\delta \left(-w^{\prime }\right)\right]}_{x_{1}=0}^{x_{1}=L}+{\left[{\bar{m}}_{r}\delta \left(-w^{\prime }\right)\right]}_{x_{1}=0}^{x_{1}=L}\;+\overset{L}{\underset{0}{\int }}{\bar{p}}_{r}{\;\delta \mathrm{Udx}}_{1}+\overset{L}{\underset{0}{\int }}{\bar{q}}_{r}{\;\delta \mathrm{wdx}}_{1}-\overset{\;}{\underset{V}{\int }}\left({E\varepsilon }_{11}{\delta \varepsilon }_{11}+\mu_{111}{\delta \;\varepsilon }_{11}^{\prime }\right)\mathrm{dV}\;-\overset{L}{\underset{0}{\int }}\left(\rho A\ddot{U}\delta U+\rho I{\ddot{w}}^{\prime }\delta w^{\prime }+\rho A\ddot{w}\delta w\right){\mathrm{dx}}_{1}-\overset{L}{\underset{0}{\int }}\left(\gamma A{\ddot{U}}^{\prime }\delta U+\gamma I{\ddot{w}}^{\prime \prime }\delta w^{\prime \prime }\right){\mathrm{dx}}_{1}=0.$$

If $\mu_{111}$ is replaced by the constitutive law (60), then this equation implies Equation (95).

## Appendix B

We observe that Equation $\left(120\right)$ can be recast with the aid of Equation (56) as follows

(A8) $$\overset{\;}{\underset{V}{\int }}\left(\partial_{1}\Sigma_{11}\right)\delta \mathrm{UdV}+\overset{\;}{\underset{V}{\int }}\left(\partial_{1}\Sigma_{11}\right)x_{3}\delta \left(-w^{\prime }\right)\mathrm{dV}+\overset{\;}{\underset{V}{\int }}\left(\partial_{3}\Sigma_{13}\right)\delta \mathrm{UdV}\;+\overset{\;}{\underset{V}{\int }}\left(\partial_{3}\Sigma_{13}\right)x_{3}\delta \left(-w^{\prime }\right)\mathrm{dV}+\overset{\;}{\underset{V}{\int }}\left(\partial_{1}\Sigma_{13}\right)\delta \mathrm{wdV}\;+\overset{\;}{\underset{V}{\int }}\left(\partial_{3}\Sigma_{33}\right)\delta \mathrm{wdV}+{\;\delta W}_{r}^{\left(\mathrm{in}\right)}=0.\;$$

The calculation of the integrals containing stress components is based on the definitions (126)–(128) and is quite similar to corresponding calculations in the last section (cf. also [25]).

The results are

(A9) $$\overset{\;}{\underset{V}{\int }}\left(\partial_{1}\Sigma_{11}\right)\delta \mathrm{UdV}={\left[N_{r}\delta U\right]}_{x_{1}=0}^{x_{1}=L}+{\left[H_{r}\delta U^{\prime }\right]}_{x_{1}=0}^{x_{1}=L}-\overset{\;}{\underset{V}{\int }}\left({E\varepsilon }_{11}\delta U^{\prime }+\mu_{111}\delta U^{\prime \prime }\right)\mathrm{dV}\;-\overset{\;}{\underset{V}{\int }}\mu_{111}\delta U^{\prime \prime }\mathrm{dV},$$

(A10) $$\overset{\;}{\underset{V}{\int }}\left(\partial_{1}\Sigma_{11}\right)x_{3}\delta \left(-w^{\prime }\right)\mathrm{dV}={\left[M_{r}\delta \left(-w^{\prime }\right)\right]}_{x_{1}=0}^{x_{1}=L}+{\left[m_{r}\delta \left(-w^{\prime \prime }\right)\right]}_{x_{1}=0}^{x_{1}=L}\;-\overset{\;}{\underset{V}{\int }}[{E\varepsilon }_{11}x_{3}\delta \left(-w^{\prime \prime }\right)+\mu_{111}x_{3}\delta \left(-w^{\prime \prime \prime }\right)\mathrm{dV},$$

(A11) $$\overset{\;}{\underset{V}{\int }}\left(\partial_{3}\Sigma_{13}\right)\delta \mathrm{UdV}=\overset{L}{\underset{0}{\int }}p_{r}{\;\delta \mathrm{Udx}}_{1}$$

(A12) $$\overset{\;}{\underset{V}{\int }}\left(\partial_{3}\Sigma_{33}\right)\delta \mathrm{wdV}+\overset{\;}{\underset{V}{\int }}\left(\partial_{3}\Sigma_{13}\right)x_{3}\delta \left(-w^{\prime }\right)\mathrm{dV}+\overset{\;}{\underset{V}{\int }}\left(\partial_{1}\Sigma_{13}\right)\delta \mathrm{wdV}=\;{\left[V_{r}\delta w\right]}_{x_{1}=0}^{x_{1}=L}+\overset{L}{\underset{0}{\int }}q_{r}{\;\delta \mathrm{wdx}}_{1}.\;$$

Substitution of these results into Equation (A8), and collecting terms, leads to

(A13) $$\begin{array}{c} {\left[N_{r}\delta U\right]}_{x_{1}=0}^{x_{1}=L}+{\left[H_{r}\delta U^{\prime }\right]}_{x_{1}=0}^{x_{1}=L}+{\left[V_{r}\delta w\right]}_{x_{1}=0}^{x_{1}=L}+{\left[M_{r}\delta \left(-w^{\prime }\right)\right]}_{x_{1}=0}^{x_{1}=L}+{\left[m_{r}\delta \left(-w^{\prime \prime }\right)\right]}_{x_{1}=0}^{x_{1}=L}\\ +\overset{L}{\underset{0}{\int }}p_{r}{\;\delta \mathrm{Udx}}_{1}+\overset{L}{\underset{0}{\int }}q_{r}{\;\delta \mathrm{wdx}}_{1}+{\delta W}_{r}^{\left(\mathrm{in}\right)}\\ =\overset{\;}{\underset{V}{\int }}\left({E\varepsilon }_{11}{\delta \varepsilon }_{11}+\mu_{111}{\delta \varepsilon }_{11}^{\prime }\right)\mathrm{dV}=\delta \Pi^{\left(i\right)}, \end{array}$$

with $\Pi_{r}^{\left(i\right)}$ being defined in Equation (98).
